# Inhibition of Intimal Thickening By PRH (Proline-Rich Homeodomain) in Mice

**DOI:** 10.1161/ATVBAHA.122.318367

**Published:** 2023-01-26

**Authors:** Lien M. Reolizo, Helen Williams, Kerry Wadey, Aleksandra Frankow, Ze Li, Kevin Gaston, Padma-Sheela Jayaraman, Jason L. Johnson, Sarah J. George

**Affiliations:** 1 Bristol Heart Institute, University of Bristol, UK (L.M.R., H.W., K.W., A.F., Z.L., J.L.J., S.J.G.).; 2 School of Medicine and Biodiscovery Institute, Faculty of Medicine & Health Sciences, University of Nottingham, UK (K.G., P.-S.J.).

**Keywords:** endothelial cell, mice, monocyte, phenotype, saphenous vein, smooth muscle

## Abstract

Late vein graft failure is caused by intimal thickening resulting from endothelial cell (EC) damage and inflammation which promotes vascular smooth muscle cell (VSMC) dedifferentiation, migration, and proliferation. Nonphosphorylatable PRH (proline-rich homeodomain) S163C:S177C offers enhanced stability and sustained antimitotic effect. Therefore, we investigated whether adenovirus-delivered PRH S163C:S177C protein attenuates intimal thickening via VSMC phenotype modification without detrimental effects on ECs.

**Methods:**

PRH S163C:S177C was expressed in vitro (human saphenous vein–VSMCs and human saphenous vein–ECs) and in vivo (ligated mouse carotid arteries) by adenoviruses. Proliferation, migration, and apoptosis were quantified and phenotype was assessed using Western blotting for contractile filament proteins and collagen gel contraction. EC inflammation was quantified using VCAM (vascular cell adhesion protein)-1, ICAM (intercellular adhesion molecule)-1, interleukin-6, and monocyte chemotactic factor-1 measurement and monocyte adhesion. Next Generation Sequencing was utilized to identify novel downstream mediators of PRH action and these and intimal thickening were investigated in vivo.

**Results:**

PRH S163C:S177C inhibited proliferation, migration, and apoptosis and promoted contractile phenotype (enhanced contractile filament proteins and collagen gel contraction) compared with virus control in human saphenous vein–VSMCs. PRH S163C:S177C expression in human saphenous vein–ECs significantly reduced apoptosis, without affecting cell proliferation and migration, while reducing TNF (tumor necrosis factor)-α–induced VCAM-1 and ICAM-1 and monocyte adhesion and suppressing interleukin-6 and monocyte chemotactic factor-1 protein levels. PRH S163C:S177C expression in ligated murine carotid arteries significantly impaired carotid artery ligation-induced neointimal proliferation and thickening without reducing endothelial coverage. Next Generation Sequencing revealed STAT-1 (signal transducer and activator of transcription 1) and HDAC-9 (histone deacetylase 9) as mediators of PRH action and was supported by in vitro and in vivo analyses.

**Conclusions:**

We observed PRH S163C:S177C attenuated VSMC proliferation, and migration and enhanced VSMC differentiation at least in part via STAT-1 and HDAC-9 signaling while promoting endothelial repair and anti-inflammatory properties. These findings highlight the potential for PRH S163C:S177C to preserve endothelial function whilst suppressing intimal thickening, and reducing late vein graft failure.

**Highlights**

- A new mechanism is revealed: PRH (proline-rich homeodomain) promotes vascular smooth muscle cell contractile phenotype and retards proliferation and migration via HDAC-9 (histone deacetylase 9) and STAT-1 (signal transducer and activator of transcription 1).
- A novel anti-inflammatory property of PRH revealed in endothelial cells.
- PRH is an attractive therapeutic target for suppressing intimal thickening and thereby late vein graft failure via the beneficial effects on vascular smooth muscle cell phenotype and behavior and endothelial cell function.

The choice of graft conduit is imperative to the success of coronary artery bypass grafts (CABG) since the patency of the conduit is an important determinant of normal postoperative outcome and patient survival.^1^ To date, the most used graft conduit is the autologous human saphenous vein (HSV), in particular, for patients with multivessel disease where 75% of grafts utilize saphenous vein.^2^ Although patients experience improved survival and symptomatic relief post-CABG surgery, long-term success is hindered by the high prevalence of vein graft failure (VGF). At 1-year postimplantation of saphenous vein grafts the occlusion prevalence is 10% to 15%.^3^ Although 5 to 10 years following CABG, implanted vein graft patency is ≈50% to 60% due to late VGF.^4^ Consequently, there is a high incidence of patients with recurrence of symptoms requiring additional interventions or repeat CABG surgery.^5^ Therefore, CABG with HSV can be considered a useful palliative treatment rather than a cure.^6^ Nonetheless, the use of venous conduits remains a fundamental requirement for CABG and are still the most frequently used owing to its length and ease of harvesting.^4^

Intimal thickening resulting from aberrant vascular smooth muscle cell (VSMC) behavior is a key component of multiple vascular pathologies including late VGF, postangioplasty restenosis, atherosclerosis, and aneurysms.^7^ The vascular injury resulting from endothelial cell (EC) damage and inflammation promotes VSMC dedifferentiation and resultant aberrant, uncontrolled migration, and proliferation of VSMCs within the intimal layer of the blood vessel wall. VSMC dedifferentiation is characterized by the switching of VSMCs from the contractile phenotype which exhibit high expression of contractile filament proteins including calponin and smoothelin to the synthetic or dedifferentiated state. Consequent intimal thickening triggers the narrowing of the lumen and causes susceptibility of the bypass graft to superimposed atherosclerosis, thrombosis, and occlusion. Therefore, there is a pressing unmet clinical need to identify new approaches that reduce VSMC migration and proliferation and intimal thickening without having detrimental effects on EC coverage and function. Unraveling ways to maintain or restore the contractile phenotype while encouraging normal EC function, is an attractive strategy for efficiently preventing vascular remodeling disorders.

The PRH (proline-rich homeodomain) protein, also known as HHEX (hematopoietically expressed homeobox), is a highly conserved transcription factor belonging to a family of proteins that serve key regulatory functions in cellular development and differentiation via both transcriptional and post-transcriptional mechanisms.^8^ Since PRH is a highly conserved transcription factor, this likely means it is evolutionarily of crucial importance and participates in many different functions. For example, adenovirus-mediated PRH overexpression in liver cancer cells attenuated hepatocarcinoma growth in a xenograft mouse model.^9^ A previous study also showed it inhibited migration of breast and prostate epithelial cells through direct transcriptional regulation of Endoglin.^10^ The PRH S163C:S177C mutated protein was mutated in the PRH homeodomain but retains DNA binding and transcriptional activities. Phosphorylation of PRH is linked to inactivation of PRH as the phosphorylated protein is rapidly degraded. PRH S163C:S177C cannot be phosphorylated by protein kinase CK2 (casein kinase 2) at these serine residues within the homeodomain resulting in a protein that has greater stability than the wild-type protein.^11,12^ Our previous study demonstrated that wild-type PRH is antiproliferative in VSMCs. Wild-type PRH expression in primary cultures of rat aortic VSMCs suppressed proliferation, whilst siRNA-induced knockdown of PRH stimulated VSMCs proliferation.^12^ Most remarkably, expression of PRH S163C:S177C allowed for a prolonged cell cycle arrest for up to 96 hours compared with wild-type PRH which was only stable for up to 24 hours.^12^ In addition, adenovirus-mediated gene delivery of PRH S163C:S177C impaired intimal thickening and VSMC proliferation in segments of HSV in vitro.^12^

In this study, we investigated whether overexpression of PRH S163C:S177C can effectively retard VSMC dedifferentiation, migration, proliferation, and intimal thickening without damaging EC function both in vitro and in vivo. Moreover, we investigated the underlying mechanisms responsible for the modulation of VSMC behavior by PRH.

## Methods

The materials and methods are outlined in detail within the Supplemental Material.

### Cell Culture

VSMCs were isolated from sections of HSV obtained from patients (Ethics number REC: 14/EE/1097) as described previously.^12^ All experiments were performed with at least four different batches of HSV-SMCs from different individuals at passage 3 to 9. HSV-ECs were purchased from Promocell (C-12231) or Stratech (HEC18-NEU-500000Ce) and grown in full EC growth medium at 37 °C, 5% CO_2_. All experiments were performed with at least 4 different batches of HSV-ECs from different individuals at passage 2 to 5. THP-1 (human leukemic cell line) cells were grown in Roswell Park Memorial Institue-1640 medium (42401018; Gibco) supplemented with 10% (v/v) heat-inactivated FBS, 2 mM L-glutamine, and 100 μg/mL penicillin and 100 IU/mL streptomycin at 37 °C, 5% CO_2_.

### Adenoviral Infection

Recombinant adenoviruses were produced to overexpress c-myc–tagged S163C:S177C mutant PRH (Ad [adenovirus]: PRH S163C:S177C) and empty adenoviral vector lacking a transgene (Ad: Control).^12^ The recombinant adenoviral constructs were depleted of ΔE1E3 by site-specific FLP (flipase)- mediated recombination to create replication incompetence. Cells were infected with Ad: PRH S163C:S177C diluted in the cell type-specific culture medium to a final concentration of 5×10^8^ plaque-forming units (pfu)/mL for VSMCs, and a combined dose of 4×10^8^ pfu/mL Ad: Control and 1×10^8^ pfu/mL for HSV-ECs. The controls for both cell types were cells infected with 5×10^8^ pfu/mL of Ad: Control or uninfected cells (media alone). Medium was refreshed after 18 hours. Transgene expression was validated using Western blotting and immunocytochemistry.

### Click-iT 5-Ethynyl-2’-Deoxyuridine Imaging

To analyze cell proliferation, cells were grown on glass coverslips in culture medium supplemented with 10 μM 5-ethynyl-2’-deoxyuridine (EdU) and EdU incorporation quantified via immunofluorescence using the Click-iT EdU Alexa Fluor 488 Imaging Kit (Molecular Probes, C10337), in accordance with manufacturer’s instructions. Coverslips were mounted on slides in ProLong Gold and DAPI (4′,6-diamidino-2-phenylindole; Invitrogen, P36931).

### Scratch Wound Assay

To analyze migration, confluent layers of cells were scratched using a 1 mL pipette tip to create two parallel straight lines, simulating a wound, then culture medium was replaced and supplemented with 2 mM hydroxyurea to inhibit proliferation as described previously.^13^ Scratch wounds were photographed using the EVOS FL Color Imaging System (Thermo Fisher Scientific, AMEFC4300) and migration quantified and expressed as the distance migrated in pixels between 0 and 24 hours.

### Immunocytochemistry

Cells seeded on glass coverslips were fixed in 3% (w/v) paraformaldehyde/PBS for 10 minutes and permeabilized with 0.2% (v/v) Triton X-100/PBS. Cells were blocked with 20% (v/v) goat serum/PBS for 30 minutes at RT and probed with primary antibody (Major Resources Table) diluted in 1% (w/v) BSA (bovine serum albumin)/PBS overnight at 4 °C or for 1 hour at 37 °C. Prolong Gold Antifade Reagent with DAPI was utilized for mounting. The cells were imaged using an Olympus BX41 microscope and Q-capture pro 6.0 software.

### Western Blotting

Cells were lysed in sodium dodecyl sulfate (SDS) lysis buffer (50 mM Tris-HCl [pH 8], 10% [v/v] glycerol, 5% [w/v] SDS) and protein quantification performed using the Micro Bicinchoninic Acid Assay Kit (23235; Thermo Scientific) in accordance with manufacturer’s instructions. The same amount of protein from whole cell lysates was separated by sodium dodecyl sulfate-polyacrylamide gel electrophoresis, followed by transfer onto Trans-Blot Mini Nitrocellulose Membranes (170-4158; Bio-Rad). The membranes were blocked with 5% nonfat milk and then incubated with primary antibodies (overnight at 4 °C, Major Resources Table) and respective secondary antibodies. The abundance of immunolabeled proteins was detected using enhanced chemiluminescence and a Bio-Rad densitometer.

### Collagen Contraction Assay

VSMC contractile capability was assessed using Cell Biolabs’ Collagen-based Contraction Assay Kit (Cell Biolabs, CBA-201) following the manufacturer’s instructions. The gels were photographed, and contraction was measured at multiple time points after collagen release using Image J. Data were expressed as the distance of contraction (arbitrary units).

### THP-1 Adhesion Assay

HSV-ECs were either incubated with 10ng/ml recombinant human TNF (tumor necrosis factor)-α (C-63721; PromoKine) or subjected to shear stress using fixed-angle platform rocker (Grant Instruments, Fisher Scientific, United Kingdom) with a maximum tilt angle of 7° at 10 oscillations per minute for 24 hours at 37 °C and 5% CO_2_. Static control group were cultured in the same incubator. THP-1 cells labeled with 10 μM calcein AM were applied to the treated HUVECs (human umbilical vein endothelial cells) for 30 minutes before washing to remove non-adherent cells. Adherent cells were photographed and quantified using the EVOS FL Colour Imaging System (Thermo Fisher Scientific; Catalogue number: AMEFC4300).

### Enzyme-Linked Immunosorbent Assay

Conditioned media was collected after 24 hours. Quantikine ELISA (enzyme-linked immunosorbent assay) kits were used to detect the following proinflammatory mediators: IL (interleukin)-6 (R&D Systems, D6050), IL-8 (R&D Systems, D8000C), and CCL2 (chemokine ligand 2)/MCP-1 (monocyte chemoattractant protein-1; R&D Systems, DCP00), in accordance with the manufacturer’s instructions. The absorbance of each well was determined at 450 nm wavelength using a GloMax Explorer Multimode Microplate Reader within 30 minutes.

### Quantitative Polymerase Chain Reaction

Total RNA was extracted and purified using the miRNeasy Mini kit (217004; Qiagen). cDNA was synthesized from 100 to 500 ng/mL purified RNA using the High-Capacity RNA-to- cDNA Kit (4387406; Applied Biosystems). Quantitative polymerase chain reaction was performed using the LightCycler 480 real-time PCR System (Roche) and LightCycler 480 SYBR Green I Master (04707516001; Roche) in combination with 1 μM primers (Table S1).

### Next-Generation Sequencing

Samples were subjected to high-throughput RNA sequencing to perform genome-wide analysis of transcriptional diversity and regulation using the TruSeq stranded mRNA kit (QIAGEN) and Illumina NextSeq 550. All analysis was performed using CLC Genomics Workbench (version 12.0.2) and CLC Genomics Server (version 11.0.2). In addition, the human genome version used was hg38 with an annotation of ENSEMBL Homo_sapiens.GRCh38.97. The samples have been run on an Illumina NextSeq 550 and the aim was to acquire an average of 30 million reads per sample. The parameters were 100 ng input RNA, 15 cycles of PCR in the fragment enrichment step, and the loading molarity used was 1.5 pM.

### Bioinformatic Analysis

Next Generation Sequencing (NGS) data were first uploaded into QIAGEN’s Ingenuity Pathway Analysis (IPA) system for core analysis. Genes with a 2-fold change and an adjusted *P* value (false discovery rate correction) <0.05 were considered as differentially expressed and were investigated by IPA as advised by the Qiagen IPA expert (Ingenuity Systems Inc, Redwood City, CA). IPA was performed to identify enriched canonical pathways, diseases, and functions, prioritize the differentially regulated genes (DEGs) identified by NGS, and categorize differentially expressed transcription factors in specific diseases and functions.

### Animals

Housing, care, and all procedures involving mice were performed in accordance with the guidelines and regulations of the University of Bristol and the United Kingdom Home Office. The investigation conforms to the Guide for the Care and Use of Laboratory Animals published by the US National Institutes of Health (Publication No. 85–23, revised 1996) and was designed in accordance with the ARRIVE guidelines (Animal Research: Reporting of In Vivo Experiments) V2.^14^

### Murine Carotid Artery Ligation

To address whether PRH S163C:S177C expression affected intimal thickening, mouse carotid artery ligation was performed. Male and female, 8-week-old C57BL/6J mice were purchased from Charles River. Mice were anesthetized by inhalation of isofluorane in 100% oxygen with 1.5 μg buprenorphine hydrochloride for analgesia (Vetergesic). The left common carotid artery was isolated, and 5-0 silk suture was tied at the bifurcation of the common carotid artery to completely occlude the artery.^15^ The exposed left carotid arteries were randomly coated with 100 μL of 30% (w/v) pluronic gel containing 1.33×10^8^ pfu of the Ad: PRH S163C:S177C (with c-myc- tag) or Ad: Control (empty virus) as previously optimized.^16^ The operator was blinded to the treatment group. Mini osmotic pumps (model 2004 Alzet) were primed with bromodeoxyuridine and inserted subcutaneously into the mice to deliver bromodeoxyuridine for the duration of the experiment and quantify proliferation. After suturing the wound, animals were housed for 28 days with standard laboratory diet (2018SX Envigo) and water available ad libitum (14 mice in each group [7 males and 7 females]). After allowing 28 days for intimal thickening to occur, mice were culled using 20 mg pentobarbital sodium (Euthatal) and the carotid arteries were dissected and fixed in 10% (v/v) formalin/PBS for 24 hours. Arteries were then transferred into PBS and stored at 4 °C until processing. In addition, the left carotid arteries were coated with 100 μL of 30% (w/v) pluronic gel containing 1.33×10^8^ pfu of the Ad: PRH S163C:S177C (with c-myc- tag) or Ad: Control in 7 mice (4 males and 3 females). After 7 days, mice were culled, carotid arteries dissected, fixed, and stored as described above. In addition to the above, additional mice were employed as unligated, sham controls for comparison with the arteries collected at 7 days after ligation. These control mice were not subjected to carotid ligation and were culled using 20 mg pentobarbital sodium.

### Histological Processing and Staining

The arteries were gently positioned upright (7-day samples) and horizontal (28-day samples) in heated molten 1.5% (w/v) agar in 10% (v/v) formalin/PBS. The agar plugs were processed using the Shandon Excelsior Tissue Processor (Thermo Electron Corporation). Three to 4 μm transverse sections (7-day samples) and longitudinal sections (28 days samples) were cut and mounted onto Superfrost Plus slides. Serial sections were collected and stained systematically after reaching the point of the open and complete lumen and length of the artery, to ensure comparison of the same region in each artery for the individual analyses. For visualization and measurement of the vessel and lesion, Elastic Van Gieson staining was performed in a Shandon Varistain 24-4 Automatic Slide Stainer (Thermo Scientific) and analyzed using ImageJ software. Immunohistochemistry and immunofluorescence were utilized to detect expression of primary antibodies (Major Resources Table). Nonimmune IgG of the same species as the primary was used as negative control in all protocols at the same concentration as the primary antibody to demonstrate the specificity of the protocol.

### Statistical Analysis

All graphical data was displayed as mean±SEM and charts show data points. Power analysis revealed 80 to >90% power to detect a change in the mean with n=4. Statistical analyses were performed using GraphPad Prism 8 and Graphpad Instat statistical software. For n<6 nonparametric analysis was performed: Mann-Whitney Test for comparison of two groups, and Kruskal-Wallis 1-way ANOVA and Dunn Test for multiple comparisons post hoc test was utilized for >2 group analyses. For n≥6 normal distribution of data was assessed using a Kolmogorov and Smirnov test for normality. Data were analyzed by Student *t* tests for 2 groups, or ANOVA and Student-Newman-Keuls Multiple Comparisons post hoc test was utilized for more than two group analyses. Friedman test was used for nonparametric repeated measures analysis. Equal variance was tested as a precondition for parametric analysis and it was confirmed that the equal variance assumption was not violated. *P*<0.05 was accepted as significantly different in all statistical tests.

## Results

### Expression of PRH S163C:S177C Attenuated VSMC Proliferation and Migration In Vitro

**Figure 1.**
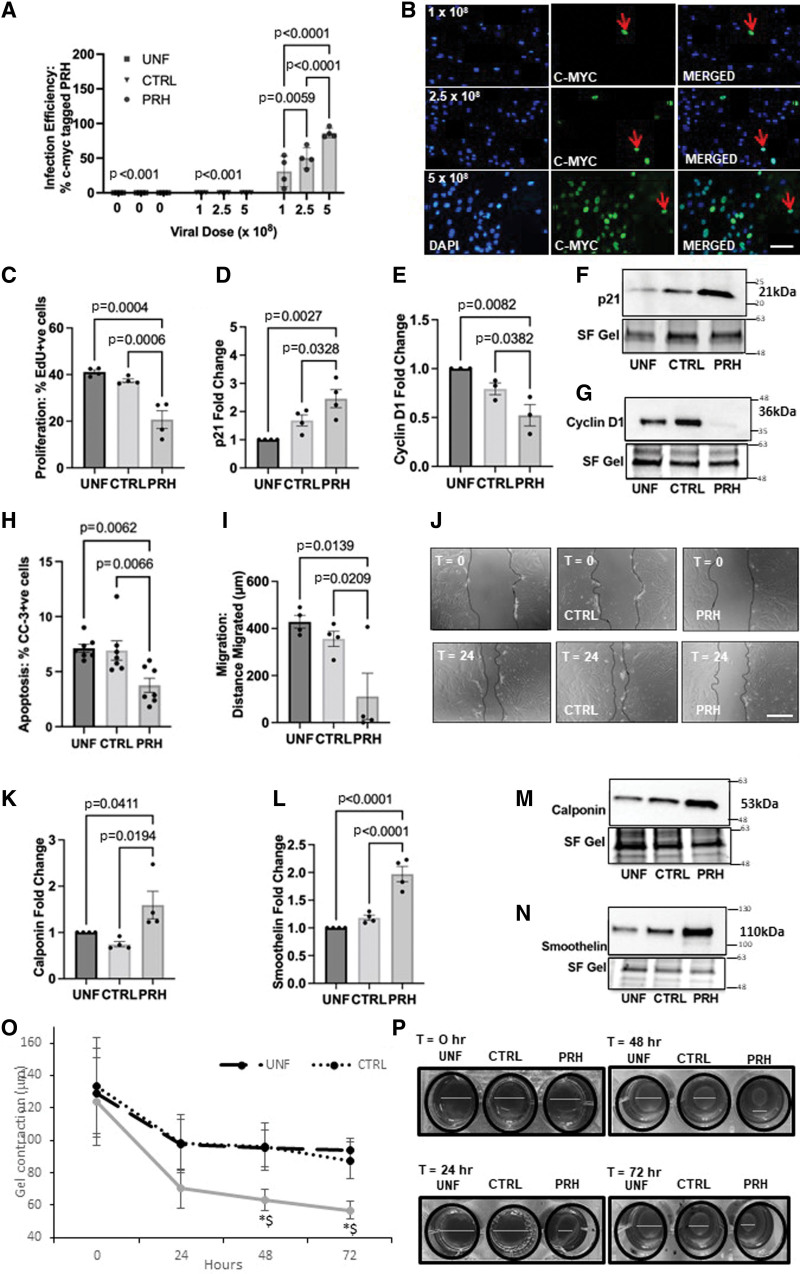
**Expression of PRH (proline-rich homeodomain) S163C:S177C attenuated vascular smooth muscle cell (VSMC) proliferation and migration while promoting the contractile phenotype in vitro. A**, Human saphenous vein (HSV) VSMCs were either infected with 1×10^8^ plaque-forming units (pfu)/mL, 2.5×10^8^ pfu/mL, or 5×10^8^ pfu/mL Ad (adenovirus): Control or Ad: PRH S163C:S177C or were left uninfected. Quantification of percentage of cells expressing c-myc–tagged PRH S163C:S177C protein. **B**, Representative images of immunocytochemistry for c-myc-tagged protein. Positive cells have green nuclei and examples are indicated with red arrows; all nuclei are stained blue with DAPI (4′,6-diamidino-2-phenylindole). Scale bar indicates 50 μm and applies to all panels. **C**, HSV-VSMCs were infected with: 5×10^8^ pfu/mL Ad: Control or Ad: PRH S163C:S177C or were left uninfected. Expression of c-myc–tagged PRH S163C:S177C in HSV-VSMCs inhibited proliferation measured as the percentage of 5-ethynyl-2’-deoxyuridine (EdU)-positive cells. Densitometric quantification of p21 protein (**D–F**) and cyclin D1 (**E–G**) expression by Western blotting; data were normalized by stain-free (SF) bands and expressed as a fold change from uninfected control. **H**, The rate of apoptosis was quantified and expressed as the percentage of cleaved caspase-3-positive cells. **I**, Migration was quantified in μm; (**J**) Representative images of scratch wound assay. Dashed line indicates wound edge. T0=0 hour; T24=24 hours. Scale bar represents 1 mm and applies to all panels. Quantification of calponin (**K**) and smoothelin (**L**) protein expression by Western blotting. Data was normalized by SF bands and expressed as a fold change from uninfected control. Representative Western blots for calponin (**M**) and smoothelin (**N**) proteins. SF bands served as a loading control. Approximate molecular weights are indicated on the right in kDa. **O** and **P**, Adenovirus-mediated delivery of Ad: PRH S163C:S177C increased contraction of VSMCs. Contraction was measured by the relative changes in collagen gel diameter. Serial changes in gel area at time 0, 24, 48, and 72 hours with an uninfected control, Ad: Control and Ad: PRH S163C:S177C, *P=0.0038 and $*P*=0.0023 vs uninfected cells (UNF) and control (CTRL), respectively. White lines indicate the gel diameter for quantification. Kruskal-Wallis ANOVA followed by Dunn multiple comparison test and Friedman test (collagen contraction), n=4 all parts except (**H**) n=7. Error bars represent SEM. CC-3 indicates cleaved caspase-3.

Firstly, the optimal adenoviral concentration to achieve high infection efficiency and expression of S163C:S177C mutant PRH (PRH S163C:S177C) in HSV-VSMCs in vitro was determined. Uninfected cells and an adenovirus without a transgene (Ad: control) served as negative controls. Immunocytochemistry revealed a significant elevation in c-myc–tagged protein expression in HSV-VSMCs infected with adenoviruses encoding PRH S163C:S177C, in comparison to the uninfected and Ad: Control (Figure 1A and 1B). The c-myc–tagged PRH S163C:S177C was localized in the nucleus. This elevation in PRH levels was confirmed by Western Blotting (Figure S1).

Quantification of EdU incorporation showed that expression of PRH S163C:S177C significantly retarded the rate of proliferation in HSV-VSMCs compared with cells infected with Ad: Control and uninfected cells (Figure 1C; Figure S1C). Additionally, HSV-VSMCs infected with adenoviruses encoding PRH S163C:S177C showed significantly elevated p21 protein levels (Figure 1D and 1F) and significantly suppressed cyclin D1 protein levels (Figure 1E and 1G) compared with uninfected cells and cells infected with Ad: Control.

Apoptosis was then quantified via immunofluorescence for CC-3 (cleaved caspase-3). As a positive control, cells were incubated with 200 ng/mL human recombinant Fas-ligand in 2% (v/v) FBS/DMEM. The level of apoptosis was similar in uninfected cells as those infected with Ad: Control with levels between 5% and 9%. Quantification of the percentage of CC-3–positive cells showed that level of apoptosis in VSMCs expressing PRH S163C:S177C was significantly lower than detected in HSV-VSMCs infected with Ad: Control or uninfected cells (Figure 1H; Figure S1D). Fas-ligand induced VSMCs apoptotic cell death as expected and acted as a positive control (Figure S1D).

The scratch wound assay was utilized to investigate the effect of PRH S163C:S177C expression on VSMC migration. Uninfected VSMCs migrated ≈400 μm (Figure 1I and 1J). In contrast, infection with Ad: Control did not significantly affect the distance migrated. The distance migrated by HSV-VSMCs infected with Ad: PRH S163C:S177C was significantly less than that observed in uninfected VSMCs and VSMCs infected with Ad: Control (Figure 1I and 1J).

### Expression of PRH S163C:S177C Increased Expression of Markers of the Contractile VSMC Phenotype

VSMCs of the contractile phenotype have high expression of smoothelin and calponin.^17^ We observed smoothelin and calponin proteins were significantly upregulated in VSMCs expressing PRH S163C:S177C compared with uninfected VSMCs and VSMCs infected with Ad: Control (Figure 1K through 1N). This finding indicates that ectopic expression of PRH S163C:S177C in VSMCs promotes the contractile and differentiated VSMC phenotype via, at least in part due to, the upregulation of smoothelin and calponin. Due to these findings, contraction was quantified using a collagen contraction assay which examines the cell-dependent contraction of collagen hydrogels. As expected, collagen gel diameter at time 0 was similar in all treatment groups and contraction was evident at each time point from 24, 48, and 72 hours in all treatment groups (Figure 1O and 1P). However, contraction was significantly greater in VSMCs infected with Ad: PRH S163C:S177C compared with the uninfected and adenovirus controls at 48 and 72 hours (Figure 1O and 1P). Taken together, these data indicate that expression of PRH S163C:S177C can inhibit VSMC proliferation and migration whilst promoting the contractile phenotype of VSMCs.

### PRH S163C:S177C Expression in HSV-ECs

**Figure 2.**
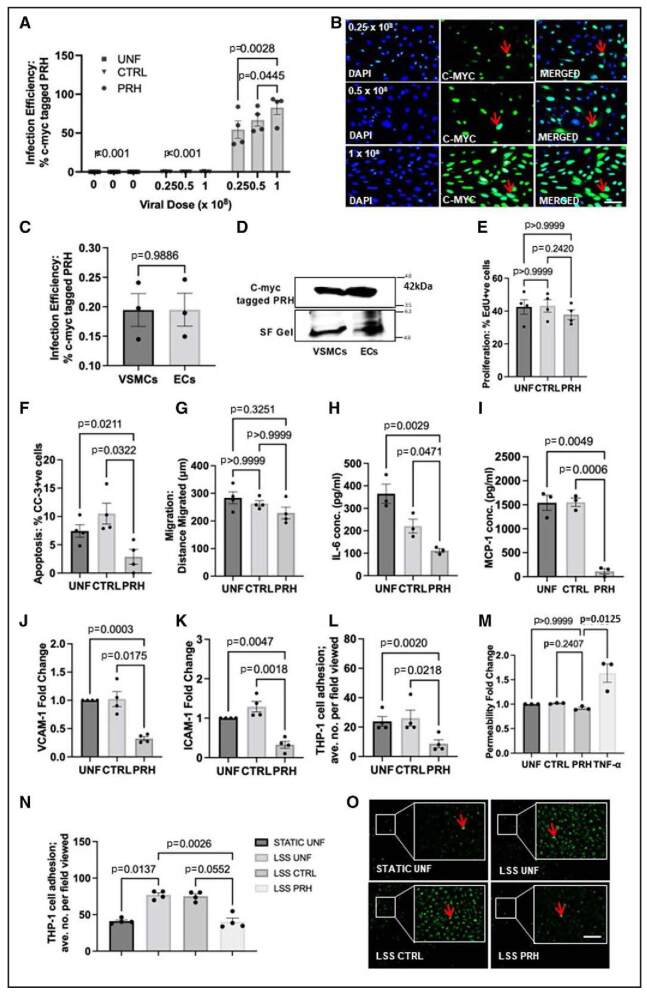
**Expression of PRH (proline-rich homeodomain) S163C:S177C exerted an anti-inflammatory role in HSV-ECs in vitro.** HSV-ECs were either infected with 1×10^8^ plaque-forming units (pfu)/mL, 2.5×10^8^ pfu/mL, or 5×10^8^ pfu/mL Ad (adenovirus): Control or Ad: PRH S163C:S177C or were left uninfected. **A**, Quantification of percentage of cells expressing c-myc–tagged PRH S163C:S177C protein. **B**, Representative images of immunocytochemistry for c-myc–tagged protein. Positive cells have green nuclei and examples are indicated with red arrows; all nuclei are stained blue with DAPI (4′,6-diamidino-2-phenylindole). Scale bar indicates 50 μm and applies to all panels. **C** and **D**, Comparable expression of c-myc–tagged PRH S163C:S177C in HSV-ECs and human saphenous vein (HSV) vascular smooth muscle cells (VSMCs). HSV-ECs were infected with 1×10^8^ pfu/mL of adenovirus encoding c-myc–tagged PRH S163C:S177C combined with 4×10^8^ pfu/mL Ad: Control. **E**, Expression of c-myc–tagged PRH S163C:S177C in HSV-ECs retarded proliferation by 5-ethynyl-2’-deoxyuridine (EdU) incorporation as the percentage of EdU-positive cells. **F**, The level of apoptosis was quantified and expressed as the percentage of CC (cleaved caspase)-3–positive cells. **G**, Migration was quantified in μm; ANOVA, Student-Newman-Keuls Multiple Comparisons Test, n=4. IL (interleukin)-6 (**H**) and MCP-1 (monocyte chemoattractant protein-1; **I**) proteins concentration (conc.) in conditioned media by ELISA; data are expressed in pg/ml. Western blotting and quantification of VCAM (vascular cell adhesion protein)-1 (**J**) and ICAM (intercellular adhesion molecule)-1 (**K**) proteins. Data were normalized using stain-free (SF) bands and expressed as a fold change from uninfected control. To assess monocyte adhesion, HSV-ECs were subsequently stimulated with TNF (tumor necrosis factor) α (**L**) for 24 hours or subjected to low wall shear stress (LSS) (**N** and **O**), then cocultured with calcein-labeled THP-1 (human leukemic cell line) cells. Monocyte adhesion was quantified and expressed as the average number of adherent cells per field viewed. Representative images showing adherent calcein-labeled THP-1 cells. Red arrow indicates some of the adherent THP-1 cell. Scale bars represent 100 µm and apply to all panels. **M**, Permeability was quantified with TNF-a positive control. Kruskal-Wallis ANOVA followed by Dunn multiple comparison test, or Mann-Whitney test, n=4. Error bars represent SEM. CTRL indicates control; EC, endothelial cell; and UNF, uninfected cells.

Comparable levels of PRH S163C:S177C protein expression were achieved between HSV-ECs and HSV-VSMCs when infected with an adenovirus encoding c-myc–tagged PRH S163C:S177C (Figure 2A through 2D; Figure S2A and S2B).

Quantification of EdU incorporation showed that the rate of proliferation in HSV-ECs expressing PRH S163C:S177C was not significantly different to that detected in HSV-ECs infected with Ad: Control and uninfected control HSV-ECs (Figure 2E; Figure S2C). The expression of cyclin D1 and p21 were not altered compared to the negative controls (Online Figure S2D). No significant effect on the distance migrated was observed between uninfected HSV-ECs or HSV-ECs infected with either Ad: Control or Ad: PRH S163C:S177C using the scratch wound assay (Figure 2; Figure S2E).

Quantification of the percentage of CC-3–positive cells showed that the level of apoptosis in HSV-ECs expressing PRH S163C:S177C was significantly lower than the level of apoptosis in HSV-ECs infected with Ad: Control or uninfected control HSV-ECs (Figure 2G; Figure S2F).

### Anti-Inflammatory Role of PRH S163C:S177C Expression in HSV-ECs

Adenovirus-mediated gene transfer of PRH S163C:S177C suppressed levels of the secreted proinflammatory mediators, IL-6 and MCP-1, with respect to both control groups (Figure 2H and 2I). Additionally, a significant reduction in the amount of adhesion molecules ICAM (intercellular adhesion molecule)-1 and VCAM (vascular cell adhesion protein)-1, which facilitates recruitment and infiltration of leukocytes into the vessel wall,^18,19^ was observed in HSV-ECs expressing PRH S163C:S177C compared with both uninfected cells and cells infected with Ad: Control (Figure 2J and 2K). Moreover, a significant reduction in the number of adherent monocytes was observed with TNFα-treated HSV-ECs expressing PRH S163C:S177C compared to control HSV-ECs (Figure 2L). Vascular permeability was not significantly enhanced by expression of PRH S163C:S177C compared to controls, whilst the positive control of TNF-α significantly increased permeability (Figure 2M). A simple rocker-induced mechanical stimulus was utilized to mimic low wall shear stress such as that observed in atheroprone regions of the vasculature exposed to disturbed flow.^20–22^ The attachment of monocytes to low wall shear stress–stimulated ECs was significantly attenuated by the expression of PRH S163C:S177C in comparison to control HSV-ECs (Figure 2N and 2O).

### Ad-Mediated Delivery of PRH S163C:S177C Retarded Intimal Thickening Without Affecting Endothelial Coverage In Vivo

**Figure 3.**
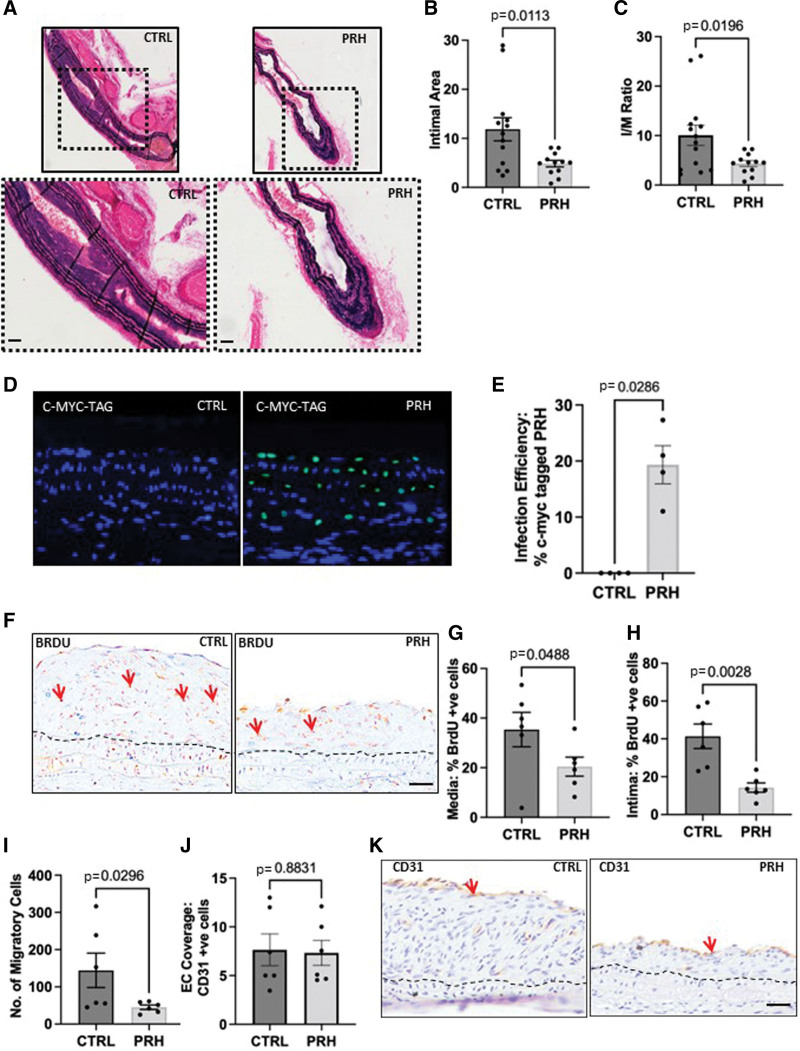
**Ad (adenovirus)-mediated delivery of PRH (proline-rich homeodomain) S163C:S177C retarded intimal thickening without affecting endothelial coverage in vivo.** Expression of PRH S163C:S177C protein attenuated neointimal area, intimal:medial (I/M) ratio, and % occlusion of the lumen. Ligated mouse carotid arteries received adventitial delivery of Ad: Control (n=13) or Ad: PRH S163C:S177C (n=13). After 28 days, the arteries were removed and subjected to Elastic Van Gieson (EVG) staining. Representative images of EVG-stained left carotid arteries (**A**) Ad: Control and Ad: PRH S163C:S177C. Scale bar represents 25 and 50 μm (**upper** and **lower**, respectively). The intimal area (**B**), I/M ratio (**C**) was measured. Representative images (**D**) and quantification (**E**) of immunofluorescence for c-myc tag at day 7. **F**, Representative images of immunohistochemistry for bromodeoxyuridine (BrdU) at day 28. Black dashed line indicates the intima-media boundary. BrdU-positive cells have brown nuclei (some are indicated with red arrows). Proliferation was quantified in the media (**G**) and intima (**H**) and expressed as the percentage of BrdU-positive cells. **I**, Migration was estimated by quantifying the number of BrdU-negative nuclei in the intima divided by the length of the intimal thickening. **J**, Endothelial coverage assessed by CD31 positive cell quantification. **K**, Representative images of immunofluorescence for CD31 28 days after ligation. CD31-positive cells are stained brown (some are indicated with red arrows); the nuclei of all cells are stained blue/gray with hematoxylin. Black dashed line indicates intimal: medial boundary. Scale bar represents 25 μm and applies to all panels. Student *t* test, unpaired, 2-tailed, n=13 (Ad: Control) and n=12 (Ad: PRH S163C:S177C) in EVG data, n=6 for the BrDU and CD31 staining. Error bars indicate SEM. CTRL indicates control; and EC, endothelial cell.

Left carotid artery ligation was performed in 8-week-old C57BL/6 mice (Charles River) to induce intimal thickening. Replication-defective adenoviral constructs encoding either an empty vector (Ad: Control) or c-myc–tagged S163C:S177C PRH was delivered peri-adventitially to the ligated arteries, which were then removed after 28 days (n=26). Immunofluorescence confirmed no expression of c-myc–tagged protein in left carotid arteries from mice infected with Ad: Control 7 days after infection, while c-myc–tagged PRH S163C:S177C protein was observed within the nuclei of cells within left carotid arteries 7 days post ligation infected with Ad: PRH S163C:S177C (Figure 3A and 3B), After 28 days, a significant reduction in intimal thickening in carotid arteries infected with Ad: PRH S163C:S177C with respect to the empty vector was observed (Figure 3C through 3F). Using our established and standard approach,^23,24^ we measured the intimal area and intimal-medial ratio which demonstrated significant reduction in the Ad: PRH S163C:S177C group compared to the empty vector controls (Figure 3D through 3F). To determine whether PRH S163C:S177C suppressed VSMC proliferation in vivo, miniosmotic pumps containing bromodeoxyuridine were implanted subcutaneously at the time of ligation. The proportion of proliferative bromodeoxyuridine-positive nuclei in the media and intima was significantly lower in the Ad: PRH S163C:S177C group than in the Ad: Control group (Figure 3G through 3I). The estimated number of migratory cells (bromodeoxyuridine-negative nuclei in the intima^13,16^) was also significantly lower in the Ad: PRH S163C:S177C group than in the Ad: Control group (Figure 3J). Sections incubated with nonimmune mouse IgG showed minimal staining demonstrating specificity of the bromodeoxyuridine protocol (Figure S3). Expression of PRH S163C:S177C did not, however, affect the cell density in both the medial and intimal layers (Figure S3B and S3C). Immunostaining for CD31 demonstrated no significant changes in the EC coverage between Ad: PRH S163C:S177C and control groups (Figure 3K and 3L). Sections incubated with nonimmune mouse IgG showed minimal staining demonstrating specificity of the CD31 protocol (Figure S3A). α-SMA (alpha-smooth muscle actin) staining confirmed the presence of VSMCs (Figure S3D), and no difference in the presence of calponin or smoothelin was noted as a result of PRH S163C:S177C expression (Online Figure S3E). Together, these data suggest that Ad: PRH S163C:S177C inhibits medial and intimal VSMC proliferation as well as migration of cells into the intima, and thereby retards intimal thickening.

### Identification of Signal Transducer and Activator of Transcription 1 and HDAC-9 as novel PRH S163C:S177C-Regulated Target Genes

In this study, NGS was employed to assist the discovery of novel PRH target genes and to determine the molecular mechanisms of the development of VGF. The first criterion used to prioritize the DEGs was to set the parameters of calculated metrics (eg, fold change, *P* value). Briefly, raw outcomes from the NGS analysis were first uploaded into QIAGEN’s IPA system for data annotation and data filtering. Two datasets were utilized in this study; Dataset A (HSV-VSMCs): Ad: Control vs Ad: PRH S163C:S177C and Dataset B (HSV-ECs): Ad: Control vs Ad: PRH S163C:S177C. Two entities of the filtered datasets were uploaded into the Compare Data tool for further filtering to obtain DEGs which are VSMC-specific and PRH S163C:S177C-regulated-specific (Figure S4A). Analysis of genes enriched in HSV-VSMCs–expressing PRH S163C:S177C were sought by subtracting genes identified in HSV-ECs.

IPA identified 2,121 DEGSs that were exclusively PRH S163C:S177C-regulated in VSMCs. However, 652 DEGs were identified as exclusively PRH S163C:S177C-regulated in HSV-ECs. There were 865 common DEGs shared between the two cell types. IPA identified canonical pathways, diseases and functions, and gene networks that are most significant to NGS outcomes and to categorize DEGs in specific diseases and functions. The differentially expressed genes were categorized into related canonical pathways based on IPA. The statistically significant enriched canonical pathways with a *P*<10^−3^ as well as representative DEGs in each canonical pathway are listed in Figure S4B. A total of 369 DEGs were identified in the Top 15 Canonical Pathways. Further stratification of DEGs was undertaken on the 369 DEGs identified. An extensive literature review by means of biological analysis was performed. As the focus of this analysis was intracellular signaling DEGs that were extracellular were eliminated as nonpriority and transcription factors or transcriptional regulators were prioritized. An in-depth literature review produced a smaller subset of potential DEG which are expressed intracellularly, has occurred in multiple pathways and had published data related to features and biological/clinical significance of VGF. Bioinformatic filtering using IPA revealed STAT-1 (signal transducer and activator of transcription 1) and HDAC-9 as PRH S163C:S177C-regulated target genes specific in VSMCs (Figure S4C) which were validated at the protein expression level.

### Pharmacologic Inhibition of STAT-1 Using Fludarabine Inhibited VSMC Proliferation

**Figure 4.**
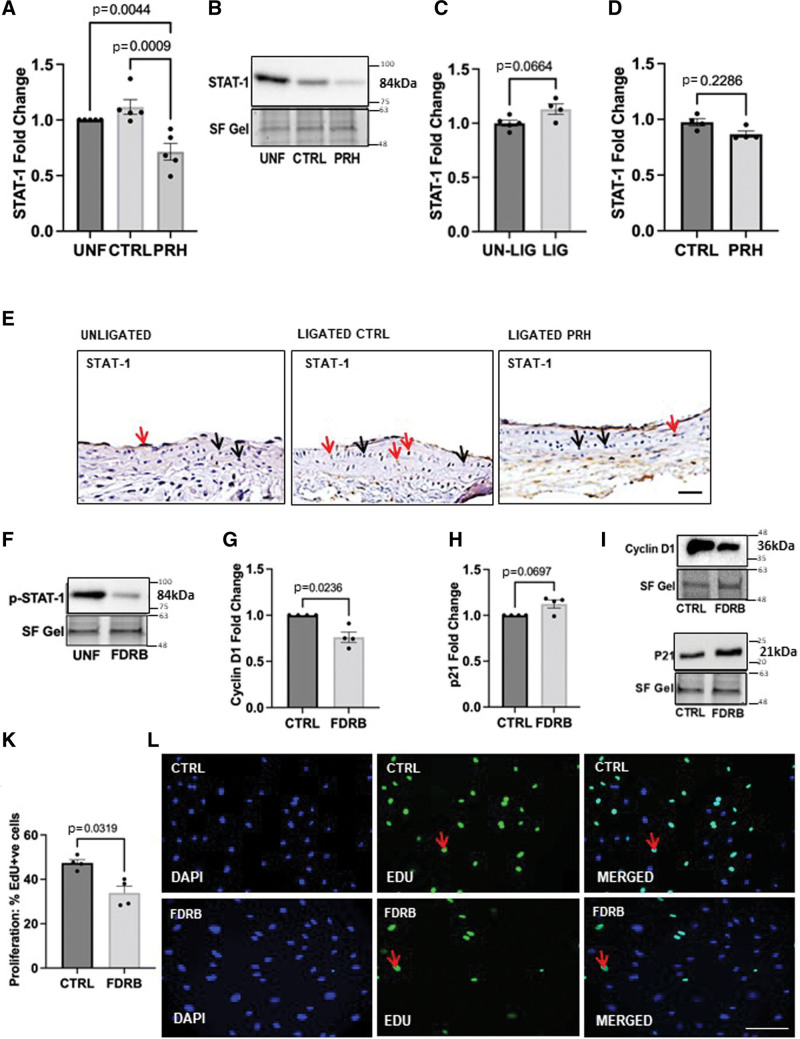
**Pharmacologic inhibition of STAT-1 (signal transducer and activator of transcription 1) using Fludarabine (FDRB) inhibited vascular smooth muscle cell (VSMC) proliferation.** Human saphenous vein (HSV) VSMCs were infected with Ad (adenovirus): Control or Ad: PRH (proline-rich homeodomain) S163C:S177C or left uninfected. **A**, Quantification of STAT-1 protein expression using Western blotting. Data were normalized using stain-free (SF) bands and expressed as a fold change from uninfected control. Kruskal-Wallis ANOVA followed by Dunn’s multiple comparison test, n=5. Error bars indicate SEM. **B**, Representative Western blot for STAT-1. SF bands served as a loading control. Approximate molecular weight of detected protein band is indicated on the right in kDa. Mouse carotid arteries were ligated in C57B/L6 mice to induce intimal thickening then subjected to infection with adenoviruses encoding either Ad: Control or Ad: PRH S163C:S177C and compared with unligated sham controls. **C**, Quantification of STAT-1 expression in ligated vs unligated mice carotids at 7 days and expressed as the fold change, Mann-Whitney test, n=4 (ligated [LIG]), n=5 (unligated [UN-LIG]). **D**, Quantification of HDAC-9 (histone deacetylase 9) protein in Ad: Control vs Ad: PRH S163C:S177C infected ligated carotid arteries at 7 days, expressed as the fold change, Mann-Whitney test, n=4. Error bars indicate SEM. **E**, Representative images of immunohistochemistry for STAT-1 protein at 7 days. Positive cells have brown nuclei (red arrow); negative cells are stained with hematoxylin and have blue nuclei (black arrow). Scale bar represents 50 μM and applies to all panels. **F**, Western blotting for p-STAT-1 (phosphorylated STAT-1) in HSV-VSMCs cultured in the presence or absence of FDRB (STAT-1 inhibitor). Densitometric quantification of cyclin D1 (**G** and **I**) and p21 protein (**H** and **J**) expression by Western blotting; data were normalized by SF bands and expressed as a fold change from uninfected control. **K**, 5-ethynyl-2’-deoxyuridine (EdU) incorporation as the percentage of EdU-positive cells in HSV-VSMCs cultured in the presence or absence of FDRB. **L**, Representative images of Click-iT EdU assay. Positive cells are green (some positive cells are indicated by red arrows), and all nuclei are stained blue with DAPI (4′,6-diamidino-2-phenylindole). Scale bar measures 50 μM and applies to all panels. CTRL indicates control; and UNF, uninfected cells.

STAT-1 was identified as one of the putative targets of PRH S163C:S177C using the bioinformatic analysis. NGS DEG gene analysis revealed STAT-1 mRNA was downregulated by 3-fold. In support of these data, Western blotting demonstrated that expression of PRH S163C:S177C reduced the protein expression of total STAT-1 in VSMCs (Figure 4A and 4B). Control unligated mouse carotid arteries and carotid arteries 7 days after ligation exhibited comparable levels of total STAT-1 positive cells (Figure 4C), and infection with Ad: PRH S163C:S177C did not significantly affect the levels of STAT-1–positive cells compared to Ad: control arteries (Figure 4D, n=4–5). Negative control sections probed with nonimmune mouse IgG showed no staining (Online Figure S3F).

To directly investigate the role of STAT-1 in VSMC proliferation, HSV-VSMCs were treated with 50 μM fludarabine (Fludarabine; STAT-1 inhibitor) or DMSO (dimethyl sulphoxide; control). The dose of 50 μM fludarabine was selected as established by previous reports,^25^ and efficacy of fludarabine inhibition of STAT-1 was demonstrated (Figure 4E). Assessment of cell growth was performed using measuring EdU incorporation 24 hours later. Treatment with 50 μM fludarabine significantly decreased VSMC proliferation compared with the DMSO control (Figure 4J and 4K) but not migration (Figure S5A and S5B or apoptosis (Figure S5C and S5D). Western blotting analysis for the cell cycle proteins, p21, and cyclin D1 revealed that fludarabine significantly decreased cyclin D1 levels (Figure 4F and 4H) but did not significantly affect p21 (Figure 4G and 4I), calponin or smoothelin (Figure S5E through S5H).

### Pharmacologic Inhibition of HDAC-9 Using TMP269 Inhibited VSMC Proliferation, Migration, and Promoted Contractile Proteins

**Figure 5.**
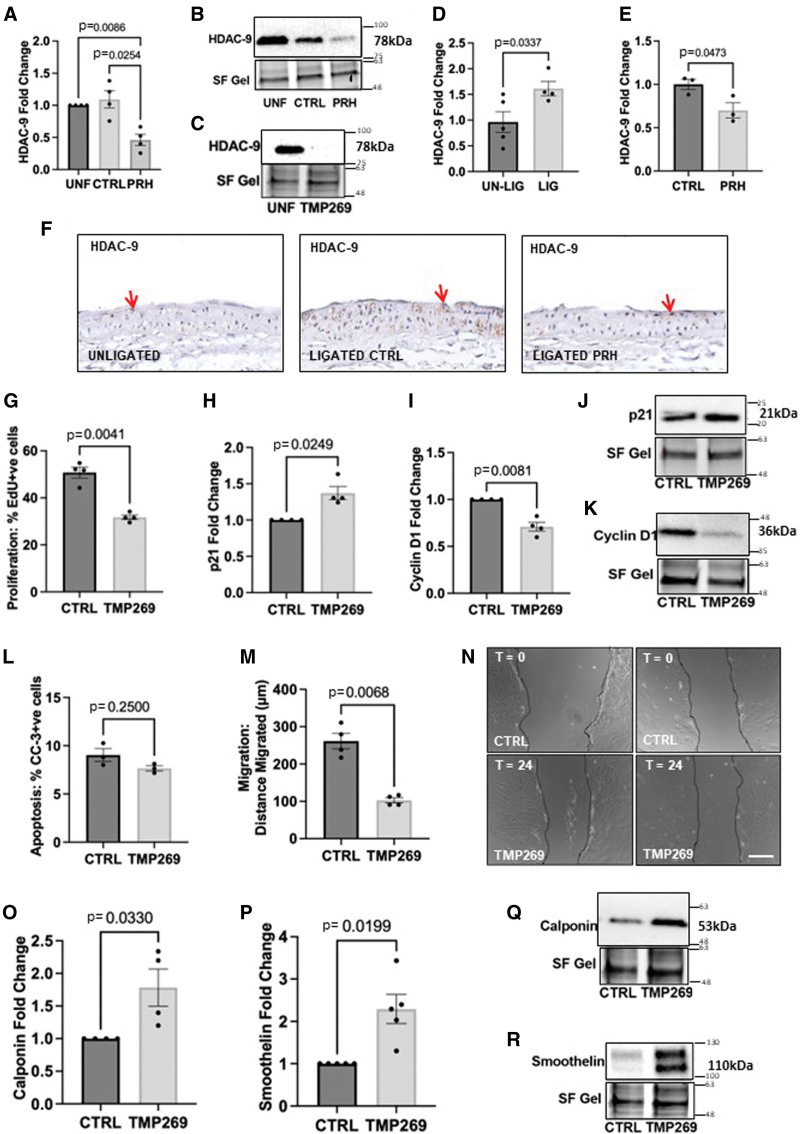
**Pharmacologic inhibition of HDAC-9 (histone deacetylase 9) using TMP269 inhibited vascular smooth muscle cell (VSMC) proliferation, migration and promoted contractile protein.** Human saphenous vein (HSV) VSMCs were infected with Ad (adenovirus): Control or Ad: S163C:S177C PRH (proline-rich homeodomain) or left uninfected. **A**, Quantification of HDAC-9 protein using Western blotting. Data were normalized using stain-free (SF) bands and expressed as a fold change from uninfected control. Kruskal-Wallis ANOVA followed by Dunn’s multiple comparison test, n=5. Error bars indicate SEM. **B**, Representative Western blot for HDAC-9. **C**, Representative Western blot for HDAC-9 in HSV-VSMCs cultured in the presence or absence of TMP269 (HDAC-9 inhibitor). SF bands served as a loading control. Approximate molecular weight of detected protein bands is indicated on the right in kDa. **D**, HDAC-9 expression in ligated vs unligated sham control mice carotids was quantified at 7 days and expressed as the fold change, Mann-Whitney test, n=4 (ligated [LIG]), n=5 (unligated [UN-LIG]). **E**, Quantification of HDAC-9 protein in Ad: Control vs Ad: PRH S163C:S177C infected ligated carotid arteries at 7 days, expressed as the fold change, Mann-Whitney test, n=3. Error bars indicate SEM. **F**, Representative images of immunohistochemistry for HDAC-9 protein at 7 days. Positive cells have brown nuclei (red arrow); negative cells are stained with hematoxylin and have blue nuclei (black arrow). Scale bar represents 50 μM and applies to all panels. **G**, 5-ethynyl-2’-deoxyuridine (EdU) incorporation as the percentage of EdU-positive cells in HSV-VSMCs cultured in the presence or absence of TMP269 (HDAC-9 inhibitor). Densitometric quantification of p21 protein (**H** and **J**) and cyclin D1 (**I** and **K**) expression by Western blotting; data were normalized by SF bands and expressed as a fold change from uninfected control. **L**, The rate of apoptosis was quantified and expressed as the percentage of CC-3 (cleaved caspase-3)–positive cells. **M**, Migration was quantified in μm. **N**, Representative images of scratch wound assay. Dashed line indicates wound edge. T0 indicates Time=0 hour; T24 indicates Time=24 hour. Scale bar represents 1 mm and applies to all panels. Mann-Whitney test, n=4. Quantification of calponin (**O**) and smoothelin (**P**) protein expression by Western blotting. Data was normalized by SF bands and expressed as a fold change from uninfected control. Representative Western blots for calponin (**Q**) and smoothelin (**R**) proteins. SF bands served as a loading control. Approximate molecular weights are indicated on the right in kDa. Mann-Whitney test, n=4. Error bars represent SEM panels. CTRL indicates control; and UNF, uninfected cells.

In addition to the discovery of STAT-1, HDAC-9 was identified as one of the putative targets of PRH S163C:S177C due to its mRNA downregulation by 3-fold. HDAC-9 protein was significantly lower in VSMCs infected with Ad: PRH S163C:S177C compared with both the uninfected VSMCs and VSMCs infected with the control virus (Figure 5A and 5B). HDAC-9 protein level was significantly elevated in the 7-day ligated left carotids compared to unligated controls (Figure 5D), and significantly downregulated in the Ad: PRH S163C:S177C group compared to control group at 28 days (Figure 5E and 5F). Negative control sections probed with nonimmune mouse IgG showed no staining (Online Figure S3F).

To investigate the role of HDAC-9 in VSMC proliferation, HSV-VSMCs were treated with 50 nM TMP269 (HDAC-9 inhibitor) in DMSO or DMSO alone (control), and efficacy of inhibition was demonstrated (Figure 5C). Treatment with 50 nM TMP269 significantly decreased VSMC proliferation compared with the DMSO control (Figure 5G; Figure S6A). Western blotting analysis for the cell cycle proteins, p21, and cyclin D1 revealed that TMP269 significantly upregulates p21 protein expression (Figure 5H and 5J) and decreases cyclin D1 levels (Figure 5I and 5K).

**Figure 6.**
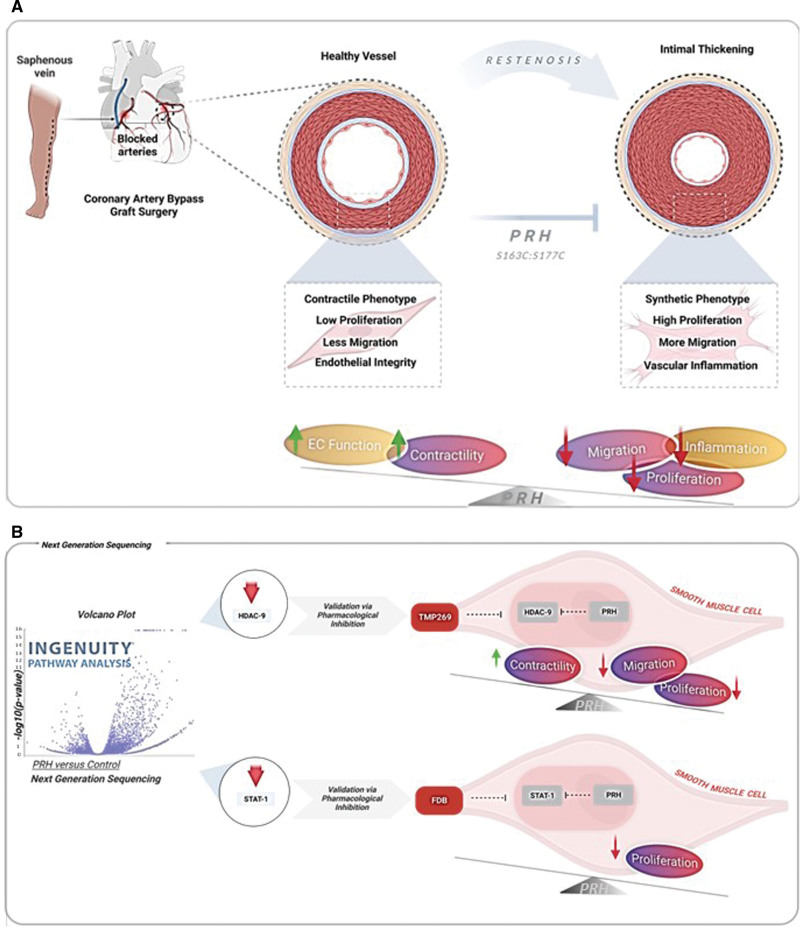
**Overexpression of PRH (proline-rich homeodomain) S163C:S177C can retard the development of intimal thickening. A**, Schematic diagram illustrating PRH S163C:S177C can effectively retard vascular smooth muscle cell (VSMC) proliferation and migration while promoting VSMC contractility without damaging endothelial function. **B**, Next Generation Sequencing (NGS) identified HDAC-9 (histone deacetylase 9) and STAT-1 (signal transducer and activator of transcription 1) as a novel PRH S163C:S177C-regulated target genes. Pharmacological inhibition of HDAC-9 using TMP269 inhibited VSMC proliferation and migration via promoting VSMC phenotypic switching from synthetic to contractile phenotype. Pharmacological inhibition of STAT-1 using Fludarabine (FDB) inhibited VSMC proliferation. Figure created from Biroender.com. EC indicates endothelial cell.

Quantification of CC-3–positive cells showed that the level of apoptosis in VSMCs was not affected with TMP269 treatment (Figure 5L and Figure 6B). Quantification of the migrated distance from the wound revealed that TMP269 significantly attenuated VSMCs migration compared to VSMCs treated with the control (Figure 5M and 5N). To investigate the role of HDAC-9 in the regulation of VSMC contractile filament proteins, the level of smoothelin and calponin proteins detected in the TMP269-treated VSMCs was significantly higher than that detected in the VSMCs treated with DMSO alone (Figure 5O through 5R).

## Discussion

This study demonstrated the ability of constitutively active, nonphosphorylatable PRH S163C:S177C mutant form to significantly reduce VSMC proliferation and migration without affecting cell death. For the first time, we demonstrated in vitro that PRH S163C:S177C enhances the contractile phenotype of VSMCs and in vivo the capability to ameliorate intimal thickening in ligated mouse carotid arteries.

Surprisingly, PRH S163C:S177C was discovered to be a novel regulator of VSMC phenotype. Previous findings by Oyama et al,^26^ demonstrated induced transcription of the SM22α and smooth muscle α-actin genes in response to wild-type PRH using rat embryonic fibroblast cells. Contrary to these findings, the present study found a pronounced increase in smoothelin and calponin protein expression following PRH S163C:S177C expression. These observations were further validated by the functional collagen contraction assay. For the first time, we have shown that cells expressing PRH S163C:S177C induced VSMC collagen gel contractile function. Conversely, no significant changes were seen in the expression of smooth muscle myosin heavy chain 10 and α-SMA (data not shown). Since expression patterns of SM22α and α-SMA occur at early stages of differentiation, while smooth muscle myosin heavy chain 10, calponin, and smoothelin genes are induced at the later stage of differentiation^27^ this implies PRH S163C:S177C results in activation of some of the late stage markers of VSMC differentiation. Collectively, the current findings show that PRH S163C:S177C promotes the differentiated, contractile VSMC phenotype in vitro.

To date, the modulation of intimal thickening by PRH S163C:S177C expression had not been investigated in vivo. We showed that PRH S163C:S177C expression suppressed aberrant proliferation of medial and intimal cells in ligated carotid arteries. As such, this implicates the success and effectiveness of the adventitial delivery of Ad: PRH S163C:S177C to reduce proliferation that results in attenuated intimal thickening. In parallel, detailed analysis revealed that migration from the media into the intima was also impaired by infection with Ad: PRH S163C:S177C. Bromodeoxyuridine-negative cells in the intima were utilized as an estimation of migratory cells, although, this way of assessing in vivo migration has been previously utilised^13,16^ it should be noted that it may underestimate migration as intimal cells may subsequently proliferate in the intima after migration. These data indicated that PRH S163C:S177C expression impaired intima development via antagonizing intimal-directed migration as well as replication. Comparably, we reported adenovirus-mediated gene transfer of PRH S163C:S177C notably disrupted intimal thickening and proliferation ex vivo implicating possible translational aspirations into humans.^12^ A previous report demonstrated the potential of an Ad-based TIMP-3 (tissue inhibitor of metalloproteinase-3) in suppressing porcine vein graft occlusion at 3 months despite lack of TIMP-3 expression at this point.^28^ Because there is a therapeutic window early after implantation, inhibiting the early detrimental remodeling via PRH could enable the graft to adapt. Hence, longer-term expression of the transgene may not be needed. Although results with Ad-TIMP-3 are promising, an additional and important benefit in the current study is the novel finding that PRH S163C:S177C promotes the differentiated contractile VSMC phenotype and contractility. Another advantage is the beneficial effect of PRH S163C:S177C on HSV-ECs. In this and our previous studies, no statistical difference in intimal area was observed when data were analyzed in a sex-specific manner and therefore data analysis was performed with combined sexes. Future studies using a porcine model, employing either an inter-positional or coronary vein graft, for up to 3 months would be beneficial to validate our findings and assess the efficacy for long-term graft patency but were beyond the scope of this current study. Data from our in vivo study illustrates that expression of PRH S163C:S177C had no detrimental effect in the endothelium compared to the control empty virus 28 days postligation. With respect to these results, data from our current study strengthens the protective effects of PRH S163C:S177C expression to the endothelium making it an ideal preventative approach to reduce VGF.

Transcriptomic profiling data obtained from NGS identified STAT-1 and HDAC-9 as downstream genes modulated by PRH S163C:S177C expression. The STAT family of 7 proteins are intracellular facilitators of cell growth, apoptosis, and lineage-specific differentiation. Fludarabine, also known as Fludara, is an established specific inhibitor of STAT-1^29^ and is a standard chemotherapy treatment for patients with chronic lymphocytic leukemia.^30^ Our findings demonstrate that STAT-1 inhibition retards VSMC proliferation and therefore may be responsible, at least in part, for the antiproliferative effects of PRH S163C:S177C. It does not, however, appear to mediate the phenotypic switching effects of PRH S163C:S177C, which may, therefore, occur through other PRH S163C:S177C-regulated targets.

HDACs are a group of chromatin-modifying enzymes that play a critical role in the regulation of gene expression. Previous genome-wide association studies have shown that genetic polymorphisms in HDAC-9 are linked to several cardiovascular diseases including coronary artery disease,^31^ atherosclerotic stroke,^32^ and atherosclerotic aortic calcification^33^ and identified as a major risk locus for vasculopathies,^34^ suggestive of a role in disease pathogenesis. Consistent with these observations, expression of HDAC-9 was upregulated in carotid atherosclerotic plaques compared to atherosclerosis-free left internal thoracic artery controls^35^ and HDAC-9 knockout mice exhibit a stable atherosclerotic plaque phenotype.^36^

The in vitro findings from the present study highlight a direct transcriptional regulation of HDAC-9 by PRH S163C:S177C. A Class IIa HDACi, TMP269 was identified as the most suitable pharmacological inhibitor due to lack of a specific HDAC-9 inhibitor. To date, there are no existing reports associating HDAC-9 and PRH. Importantly, this study suggested TMP269 can inhibit VSMC proliferation, and, for the first time, it was shown that it also retards migration of HSV-VSMCs, mirroring the effects observed with PRH S163C:S177C expression. Further analysis showed that treatment with TMP269 considerably increased the SMC-specific contractile markers calponin and smoothelin which again was comparable to the data observed from PRH S163C:S177C expression. Hence, providing a pathway by which PRH S163C:S177C modulates the VSMC proliferation, migration, and differentiation to the contractile phenotype. These data offer the first mechanistic link between PRH and HDAC-9 which could be involved in smooth muscle phenotype and thereby vascular pathologies. From the evidence generated, it is indicated that blocking the HDAC-9 pathway may be the best candidate to reduce diseases related to smooth muscle phenotypic switching within the vasculature including vein graft disease, atherosclerosis, abdominal aortic aneurysm, and pulmonary arterial hypertension, however, future studies are needed to verify this point of view.

In designing therapies for vein graft disease, it is essential that the intervention does not exacerbate EC loss or impair regrowth of endothelium to prevent postoperative thrombotic occlusion and eventual failure of the vein graft. Interestingly, in contrast to the effects observed in VSMCs, previous studies have revealed plasmid-delivered overexpression of wild-type PRH did not significantly affect cell cycle progression, cell motility, or cell survival indices in HSV-ECs in vitro.^12^ It was, therefore, important to determine whether similar effects were observed with PRH S163C:S177C. This was the case and it was observed that expression of PRH S163C:S177C in HSV-ECs via adenoviral delivery did not significantly affect endothelial proliferation, migration, or apoptosis.

The augmentation of cytokines, chemo-attractants, and cell adhesion molecules has been increasingly recognized as key indicators of disturbed endothelial function.^37^ Following implantation into the arterial circulation system, the implanted vein graft undergoes hemodynamic adaptation that promotes graft stenosis.^38,39^ At the site of the end-to-side anastomosis and venous valves, low wall shear stress on the endothelia-blood interface facilitates EC activation and dysfunction, recruitment of circulating inflammatory cells in response to release of cytokines including TNFα and expression of adhesion molecules, platelet aggregation and stimulation of VSMC proliferation.^40,41^

As observed with TNFα-stimulation and under low wall shear stress culture conditions, there was decreased monocyte adhesion to HSV-ECs expressing PRH S163C:S177C. Since EC activation is mediated by combinatorial factors, this current study selected to investigate the effects of PRH S163C:S177C on the endothelial expression of adhesion molecules, VCAM-1 and ICAM-1 as these are recognized to play a critical role in mediating firm adhesion of circulating leukocyte into the vascular endothelium under pathological conditions.^42^ However, well-known markers of inflammation, IL-6, and MCP-1 were selected to be examined owing to their recognized proinflammatory role in VGF.^43,44^ In response to PRH S163C:S177C expression, these 2 inflammatory cytokines, and two adhesion molecules were significantly downregulated in HSV-ECs. Thus, validating a novel, anti-inflammatory role for PRH S163C:S177C. It is important to note that since PRH phosphorylation is known to be important in multiple cell types, this work also has important implications for the emerging role of highly conserved serine/threonine kinase Protein Kinase CK2 in immunity.^45^ Under normal circumstances, the quiescent endothelium protects the vascular tree by mediating endothelial permeability.^38^ In this study, following confirmation of an anti-inflammatory response to PRH S163C:S177C in cultured HSV-ECs, endothelial permeability was examined in HSV-ECs and showed no significant differences across all conditions.

### Limitations

Carotid artery ligation in mice is an advantageous model to induce intimal thickening. However, several inherent limitations must be acknowledged regarding the clinical extrapolation of findings. Firstly, it is not a model of vein grafting. However, in the mouse model of vein grafting the vein is retrieved from one mouse and implanted into the artery of the recipient mouse, using two mice per graft and therefore not autologous tissue and raising ethical and cost implications. Concerningly the venous cells die in the first few days of implantation, resulting in an acellular venous scaffold that becomes repopulated by circulating cells and tissue recipient cells.^46^ This is not representative of the human intimal thickening process of graft failure. Consequently, the mouse carotid artery ligation model, a well-established model to study VSMC migration and proliferation that leads to intimal thickening involves the processes that drive human disease.

Another limitation to consider is that the model features an intact endothelium which does not mimic clinical conditions observed in CABG patients where graft harvesting, distension, and implantation causes endothelial damage and denudation.^47^ Thirdly, the effect of macrophages and vascular inflammation is difficult to analyze using this model. To overcome these shortcomings, future studies should utilize additional animal models. The porcine autologous saphenous vein–to–carotid artery interposition grafts is highly suitable.^28^ Although the potential benefits of porcine model include the ongoing issues of its technical complexity and high cost. Therefore, the mouse carotid artery ligation model remains to be a robust and established preclinical model which permits substantial understanding of adenoviral-mediated gene therapy of restenosis in vein graft degeneration.

An additional limitation of the current study was the focus on the transcription factors within VSMC-specific regulated DEGs due to the limited time to follow up on all of the regulated genes from NGS. Evidence presented in this study suggests the anti-inflammatory role of PRH S163C:S177C in ECs. Therefore, future work on the analysis of HSV-EC-specific regulated DEGs from NGS would be optimal to provide a mechanistic link on the effect of PRH S163C:S177C in the endothelium.

### Conclusions

Our evidence suggests that PRH S163C:S177C may reduce VGF by inhibiting intimal thickening through modulation of VSMC phenotype and behavior at least in part mediated by STAT-1 and HDAC-9, and reduction of endothelial inflammation. In a clinical setting, a perioperative intervention would present a unique therapeutic window of opportunity to directly deliver Ad: PRH S163C:S177C to the vein graft wall before the venous graft implantation. Together the data presented in this study clearly demonstrate the benefit of an adenoviral-based expression of the mutant and stabilized form PRH (S163C:S177C). Hence, a clinically applicable strategy for delivering PRH S163C:S177C could limit the requirement for repeat revascularisation procedures in patients undertaking arterial reconstruction. Alternatively, the utilization of pharmacological inhibitors of STAT-1 and HDAC-9 may be beneficial for the suppression of late VGF.

## Article Information

### Acknowledgments

We thank Dr Graciela Sala-Newby and Tom Hathway for assistance in preparing the adenoviruses used in this study.

### Sources of Funding

This work was supported by the British Heart Foundation (FS/17/46/33121).

### Disclosures

None.

### Supplemental Material

Supplemental Methods

Major Resources Table

Table S1

Figures S1–S9

References: 10,11,13

## Supplementary Material
