# Supplementary material for: Inhibition of Intimal Thickening By PRH (Proline-Rich Homeodomain) in Mice
Source: Arterioscler Thromb Vasc Biol. 2023 Jan 26;43(3):456–73. doi: 10.1161/ATVBAHA.122.318367 (PMC9944393; doi:10.1161/ATVBAHA.122.318367)
Supplement: Supplementary file 1 [file atv-43-456-s001.pdf]

Supplemental Materials

Inhibition Of Intimal Thickening By Proline Rich Homeodomain (PRH) in Mice

LM Reolizo[1], H Williams[1], K Wadey[1], A Frankow[1], Z Li[1], K Gaston[2], P-S Jayaraman[2], J Johnson[1], SJ George[1].

[1]Bristol Heart Institute, University of Bristol, Research Floor Level 7, Bristol Royal Infirmary, Bristol BS2 8HW.

[2]School of Medicine and Biodiscovery Institute, Faculty of Medicine & Health Sciences, University of Nottingham, Nottingham, NG7 2RD.

Running title: Inhibition of intimal thickening by PRH

Keywords: VSMC, phenotype, intimal thickening

Expanded Materials and Methods

Cell culture

HSV-VSMCs were grown in Dulbecco's Modified Eagle Medium (DMEM, Sigma, D5546) supplemented with 10% foetal bovine serum (FBS: Gibco, A15-151), 2mM L-glutamine (M11-004), 100μg/mL penicillin and 100μg/mL streptomycin (P11-010), and 8μg/ml gentamicin (P11-004) were acquired from PAA Laboratories (10% (v/v) FBS/DMEM) at 37°C, 5% CO_2.

Human saphenous vein endothelial cells (HSV-ECs) were grown in full endothelial cell growth medium (Promocell, C-22010 or Stratech, EGK001-NEU-500 ml) at 37°C, 5% CO_2. HSV-ECs were grown following the suppliers' protocols using Stratech growth media, wells were coated in AlphaBioCoat Solution (Stratech, AC001-NEU-20 ml) prior to cell seeding, or Endothelial Cell Basal Medium (Promocell, C-22210) supplemented 2% (v/v) Foetal Bovine Serum (FBS).

Immunocytochemistry

To detect bound cleaved caspase-3 antibody, cells were incubated with biotinylated goat anti-rabbit antibody diluted 1:200 in 1% (w/v) BSA/PBS for 45min, then Dylight 488/594 Streptavidin (Vector Laboratories, SA-5488-1/SA-5594-1) diluted 1:200 in 1% (w/v) BSA/PBS for 45min. Positive cells were identified as those with green-coloured nuclei whereas negative cells were those with only blue-coloured (DAPI) nuclei. To determine the proliferative rate per condition, the percentage of positive cells was calculated. A total of 20 images were taken in 20x magnification per sample (approximately 200-400 cells per sample). The number of EdU-positive cells was expressed as a percentage of the total number of cells counted.

Western Blotting

One volume Laemmli Sample Buffer (Bio-Rad, 161-0737) with 5% (v/v) β-mercaptoethanol was added to 5-10 μl of lysate, for which protein concentration was normalised with HPLC water. Samples and BLUeye Prestained Protein Ladder (Geneflow, S6-0024) were heated at 95 °C for 5 min and electrophoresed on 4-12% Mini-PROTEAN TGX Gels (Bio-Rad, catalogue number: 456-1084) using 1X Tris/Glycine/SDS (TGS) running buffer (Bio-Rad, catalogue number, 161- 0772) and

300 V for 15-20 minutes. Protein was normalised against stain-free gel bands visualised via a 1 min exposure to UV light. Proteins were transferred to Trans-Blot Mini Nitrocellulose Membranes (Bio-Rad, catalogue numbers, 170-4158) using the Trans-Blot Turbo Transfer Starter System (Bio-Rad), following the manufacturer's instructions.

Nitrocellulose membranes were blocked in 5% (w/v) fat-free milk powder diluted in Tris-buffered saline-Tween (TBST: 20mM Tris, 137mM NaCl, 0.1% (v/v) Tween 20; pH 7.6) for 30 minutes at room temperature (RT). Membranes were incubated overnight at 4°C with diluted primary antibody, washed with TBST, and incubated with appropriate HRP-conjugated secondary antibody. Following incubation, membranes were washed with TBST and incubated with Luminata Forte Western HRP substrate (Merck Millipore, WBLUF0100) for detection.
Representative Western blots are shown in Supplementary Figures VII, VIII and IX.

Collagen Contraction Assay

VSMC contractile capability was assessed using Cell Biolabs' Collagen-based Contraction Assay Kit (Cell Biolabs, CBA-201) following the manufacturer's instructions. Briefly, VSMCs were seeded into tissue culture wells and infected with recombinant adenoviruses for 18h. VSMCs were detached using 0.05% (w/v) Trypsin- Ethylenediaminetetraacetic acid (EDTA) at 37°C for 5 minutes and resuspended in 10% (v/v) FBS/DMEM. Cold collagen gel working solution was prepared by mixing the following components in this order collagen solution, 5 x PBS and neutralizing solution. The collagen gel working solution and VSMCs in suspension were mixed on ice at a ratio of 1:4. Following this, 500 µl of the final mixture was cast into each well of a 24-well culture plate. The solution was then allowed to polymerize for 30 min at RT, then 1.0 ml of culture medium was added atop each collagen gel lattice. After polymerization for 48 hours, the gels were gently released from the plates using a sterile needle to allow contraction.

qPCR

Relative quantification of mRNA was conducted using the comparative $2^{-\Delta\Delta Ct}$ method with GAPDH and 36B4 as the reference gene, in which $\Delta Ct = Ct(test) -$

Ct(control), ΔΔCt = ΔCt(Target gene)− ΔCt(GAPDH/36B4). The quality of qPCR was demonstrated by the detection of a single peak melt curve corresponding to single PCR product.

Next Generation Sequencing

HSV-VSMCs were subjected to infection with 5 x 10^8 pfu/ml of adenovirus encoding either empty vector or c-myc-tagged PRH S163C:S177C, for 18 hours. HSV-ECs were subjected to infection with 1 x 10^8 pfu/ml of adenovirus encoding c-myc-tagged PRH S163C:S177C combined with 4 x 10^8 pfu/ml of adenovirus encoding empty vector or 5 x 10^8 pfu/ml of adenovirus encoding 5 x 10^8 pfu/ml empty vector, for 18 hours. Subsequently, cells were incubated for another 24 hours after replenishing the culture medium. Total RNA was extracted, purified, and quantified prior to NGS by QIAGEN Genomic Services (Germany). Samples were subjected to high-throughput RNA sequencing to perform genome wide analysis of transcriptional diversity and regulation using the TruSeq stranded mRNA kit (QIAGEN) and Illumina NextSeq 550. All analysis was performed using CLC Genomics Workbench (version 12.0.2) and CLC Genomics Server (version 11.0.2). In addition, the human genome version used was hg38 with an annotation of ENSEMBL Homo_sapiens.GRCh38.97. The samples have been run on an Illumina NextSeq 550 and the aim was to acquire an average of 30 million reads per sample. The parameters were: 100 ng input RNA, 15 cycles of PCR in the fragment enrichment step and the loading molarity used was 1.5 pM.

Bioinformatic Analysis

IPA was performed to identify enriched canonical pathways, diseases and functions, and prioritise the differentially regulated genes (DEGs) identified by NGS and to categorize differentially expressed transcription factors in specific diseases and functions. For an in-depth literature retrieval, ‘vascular smooth muscle cell’, ‘cell proliferation’, ‘cell contractility’, ‘phenotypic switching’, ‘cell migration’, ‘endothelial cell’, and ‘inflammation’ was used as the keywords to search for the association of DEGs to vein graft failure.

Major Resources Table

Animals (in vivo studies)

Species	Vendor or Source	Background Strain	Sex	Persistent ID / URL
Mouse	Charles River	C57BL/6J	M/F	C57BL/6 Mice | Charles River (criver.com)

Antibodies used for Western Blotting

Primary antibodies were diluted in 1% (w/v) bovine serum albumin (BSA) / phosphate buffered saline (PBS), 5% (w/v) BSA/PBS, 5% (w/v) BSA/ tris-buffered saline (TBS), 5% (w/v) BSA/ tris-buffered saline-tween (TBS-T) or 5% (w/v) skimmed milk powder in TBS-T (5% milk/TBS-T) depending on manufacturer's instructions or in-house optimisation. *Secondary antibodies were diluted in PBS, 5% (w/v) BSA/TBS-T or 5% milk/TBS-T depending on manufacturer's instructions or in-house optimisation.

Target antigen	Vendor or Source	Catalog #	Working conc.	Persistent ID / URL
Calponin	Novus Biological	NBP1-87029	0.1µg/ml	Calponin 1 Antibody (NBP1-87029): Novus Biologicals
C-myc tag	Cell Signalling	2276	0.56µg/ml	Myc-Tag (9B11) Mouse mAb | Cell Signaling Technology
Cyclin D1	Cell Signalling	2978	0.1µg/ml	Cyclin D1 (92G2) Rabbit mAb | Cell Signaling Technology
HDAC-9	Abcam	Ab239979	0.5µg/mL	Recombinant Anti-HDAC9 antibody [EPR5223] - BSA and Azide free KO Tested (ab239979) (abcam.com)
ICAM-1	Abcam	Ab53013	0.3µg/mL	Recombinant Anti-ICAM1 antibody [EP1442Y]

				(ab53013) | Abcam
MYH11	Novus Bio	NBP2-47900	0.1µg/mL	Myosin heavy chain 11 Antibody (MYH11/923) - Azide and BSA Free (NBP2-47900): Novus Biologicals
P21	Cell Signalling	2947	0.1µg/mL	p21 Waf1/Cip1 (12D1) Rabbit mAb | Cell Signaling Technology
Smoothelin	Novus Biological	NBP2-37971	0.5µg/mL	Smoothelin Antibody (NBP2-37971): Novus Biologicals
STAT-1	Cell Signalling	14994S	0.1µg/mL	Stat1 (D1K9Y) Rabbit mAb | Cell Signaling Technology
p-STAT-1	Cell Signalling	9167S	0.1µg/mL	Phospho-Stat1 (Tyr701) (58D6) Rabbit mAb | Cell Signaling Technology
VCAM-1	Abcam	Ab134047	0.44µg/mL	Recombinant Anti-VCAM1 antibody [EPR5047] KO Tested (ab134047) | Abcam
*Anti- Mouse IgG-HRP	Dako	P0260-2	1:2000 dilution	P026002-2 | Agilent
*Anti- Mouse IgG-HRP	Cell Signalling	7076	1:2000 dilution	Anti-mouse IgG, HRP-linked Antibody | Cell Signaling Technology
*Anti-Rabbit IgG-HRP	Dako	P0217-2	1:5000 dilution	P021702-2 | Agilent

*Anti-Rabbit IgG-HRP	Cell Signalling	7074	1:2000 dilution	Anti-rabbit IgG, HRP-linked Antibody | Cell Signaling Technology

Abbreviations. HRP: Horseradish Peroxidase; ICAM-1: Intercellular Adhesion Molecule 1; HDAC-9: Histone Deacetylase 9; STAT-1: Signal Transducer And Activator Of Transcription 1; VCAM-1: Vascular cell adhesion protein; IgG: Immunoglobulin-G; WB: Western Blotting.

Antibodies used for Immunocytochemistry, Immunohistochemistry, and Immunofluorescence

Primary antibodies were diluted in 2-5% (w/v) bovine serum albumin (BSA) / phosphate buffered saline (PBS), 5% (w/v) BSA/PBS, depending on manufacturer's instructions or in-house optimisation. *Secondary and Tertiary antibodies were diluted in PBS or 5% (w/v) BSA/PBS depending on manufacturer's instructions or in-house optimisation.

Target antigen	Vendor or Source	Catalog #	Working conc.	Persistent ID / URL
Cleaved Caspase-3	R & D systems	AF835	ICC: 1µg/ml	Human/Mouse Active Caspase-3 Antibody AF835: R&D Systems (rndsystems.com)
Myc tag	Millipore	16224	ICC: 4µg/ml	Anti-Myc Tag Antibody, clone 4A6, Alexa Fluor® 488 conjugate | 16-224 (merckmillipore.com)
α Smooth Muscle Cell Actin	Sigma Aldrich	A2547	IF: 3.1µg/ml	Monoclonal Anti-Actin, a-Smooth Muscle clone 1A4, ascites fluid Anti-Alpha Smooth Muscle Actin Antibody (sigmaaldrich.com)
BrdU	Sigma Aldrich	B2531	IHC: 8.6µg/mL	BrdU monoclonal antibody Brdu Antibody (sigmaaldrich.com)
C-Myc-Tag	Cell Signalling	2276	IHC: 22.4ng/mL	Myc-Tag (9B11) Mouse mAb | Cell Signaling Technology
Calponin	Novus Biologicals	NBP1-87029	IHC: 0.2ug/mL	Calponin 1 Antibody (NBP1-87029): Novus Biologicals

CD31	Abcam	Ab28364	IHC: 0.4µg/mL	Anti-CD31 antibody (ab28364) | Abcam
HDAC-9	Abcam	Ab239979	IHC: 1µg/mL	Recombinant Anti-HDAC9 antibody [EPR5223] - BSA and Azide free KO Tested (ab239979) (abcam.com)
Smoothelin	Novus Biological	NBP2-37971	IHC: 1µg/mL	Smoothelin Antibody (NBP2-37971): Novus Biologicals
STAT-1	Cell Signalling	14994S	IHC: 1µg/mL	Stat1 (D1K9Y) Rabbit mAb | Cell Signaling Technology
* AlexaFluor-488 goat anti-rabbit IgG	Invitrogen	A11008	1:2000 dilution	Goat anti-Rabbit IgG (H+L) Cross-Adsorbed, Alexa Fluor® 488 (A-11008) (thermofisher.com)
* DyLight-488 goat anti-rabbit IgG	Vector	DI-1488	1:2000 dilution	Anti Rabbit IgG, DyLight 488 conjugated, made in goat x rabbit (vectorlabs.com)
* Biotinylated goat anti-mouse IgG	Vector	BA-9200	1:2000 dilution	Anti Mouse IgG (H+L), made in goat, Biotinylated x mouse (vectorlabs.com)
* Biotinylated goat anti-rabbit IgG	Sigma Aldrich	B7389	1:2000 dilution	Anti-Rabbit IgG (whole molecule) - Biotin antibody produced in goat, form - buffered aqueous solution (sigmaaldrich.com)
* Dylight-488 Streptavidin	Vector	SA-5488	1:2000 dilution	Streptavidin, DyLight 488 Conjugated (vectorlabs.com)
*ExtrAvidin-peroxidase	Sigma Aldrich	E2886	1:2000 dilution	ExtrAvidin®–Peroxidase buffered aqueous solution | Sigma-Aldrich (sigmaaldrich.com)

Abbreviations. HRP: Horseradish Peroxidase; ICC: Immunocytochemistry; IF: Immunofluorescence; IHC: Immunohistochemistry; BrdU: 5-bromo-2'-deoxyuridine; CD31: Cluster of Differentiation 31; HDAC-9: Histone Deacetylase 9; STAT-1: Signal Transducer And Activator Of Transcription 1; IgG: Immunoglobulin-G.

Cultured Cells

Species	Vendor or Source	Catalog #	Sex	Persistent ID / URL
Human saphenous vein VSMCs	Isolated in house from consenting donors	N/A	Male and female	N/A
Human saphenous vein endothelial cells	Promocell	C-12231	Male and female	Human Saphenous Vein Endothelial Cells (HSaVEC) - PromoCell
Human saphenous vein endothelial cells	Stratech	HEC18-NEU-500000Ce	Male and female	Human Saphenous Vein Endothelial Cells - Stratech
Human THP-1 cells	ATCC	TIB-202	unknown	THP-1 | ATCC

ARRIVE GUIDELINES

The ARRIVE guidelines (https://arriveguidelines.org/) are a checklist of recommendations to improve the reporting of research involving animals. Key elements of the study design should be included below to better enable readers to scrutinize the research adequately, evaluate its methodological rigor, and reproduce the methods or findings.

Study Design

**Groups**	**Sex**	**Age**	**Number (prior to experiment)**	**Number (after termination)**	**Littermates (Yes/No)**	**Other description**
Group 1 (Ad.Control) 28 day ligations	Male	8weeks	7	7	Yes	
Group 1 (Ad.Control) 28 day ligations	Female	8weeks	7	7	Yes	
Group 2 (Ad.PRH S163C:S177C) 28 day ligations	Male	8weeks	7	7	Yes	
Group 2 (Ad.PRH S163C:S177C) 28 day ligations	Female	8weeks	7	7	Yes	
Group 1 (Ad.Control) 7 day ligations	Male	8weeks	4	4	Yes	
Group 1 (Ad.Control) 7 day ligations	Male	8weeks	3	3	Yes	
Group 2 (Ad.PRH S163C:S177C) 28 day ligations	Male	8weeks	4	4	Yes	
Group 2 (Ad.PRH S163C:S177C) 28 day ligations	Female	8weeks	3	3	Yes	

Sample Size: Please explain how the sample size was decided Please provide details of any a *prior* sample size calculation, if done.

A sample size of 7 per group was used for day 7 ligation studies, providing 90% power to detect a 50% change in protein expression. A sample size of 14 per group was used for day 14 ligation studies, providing 95% power to detect a 50% reduction in intima size. These calculations were

performed using ‘ClinCalc’ and our previously published studies using the carotid artery ligation model (intimal area 12±4.9, protein expression 25±7.5: mean±SD).

Inclusion Criteria

All mice

Exclusion Criteria

None

Randomization

Mice randomly allocated to treatment groups

Blinding

Operator blinded to treatment group at time of adenovirus delivery and during all subsequent histological analysis and data unblinded after statistical analysis completed.

Online Table S1

GENE	PRIMER	SEQUENCE OR CATALOGUE NUMBER	COMPANY
**ICAM-1**	ICAM-1	FORWARD: GCAGACAGTGACCATCTACAGCTT REVERSE: GCCTCACACTTCACTGTCACCTC	SIGMA
**VCAM-1**	VCAM-1	FORWARD: ACTTGATGTTCAAGGAAGAG REVERSE: TCCAGTTGAACATATCAAGC	SIGMA
**SMTN**	Smoothelin	QT00026677	QIAGEN
**CNN1**	Calponin	QT00067718	QIAGEN
**IL-6**	Interleukin-6	FORWARD: AAATTCGGTACATCCTCGACGGCA REVERSE: TTTTCACCAGGCAAGTCTCCTCAT	SIGMA
**IL-8**	Interleukin-8	FORWARD: TGTGAAGGTGCAGTTTTGCCAAGG REVERSE: AATTTCTGTGTTGGCGCAGTGTGG	SIGMA
**MCP-1**	Monocyte Chemotactic Protein-1	FORWARD: CTCAGCCAGATGCAATCAATGCCC REVERSE: TTCTTTGGGACACTTGCTGCTGGT	SIGMA

Supplemental Online Figures S1-9

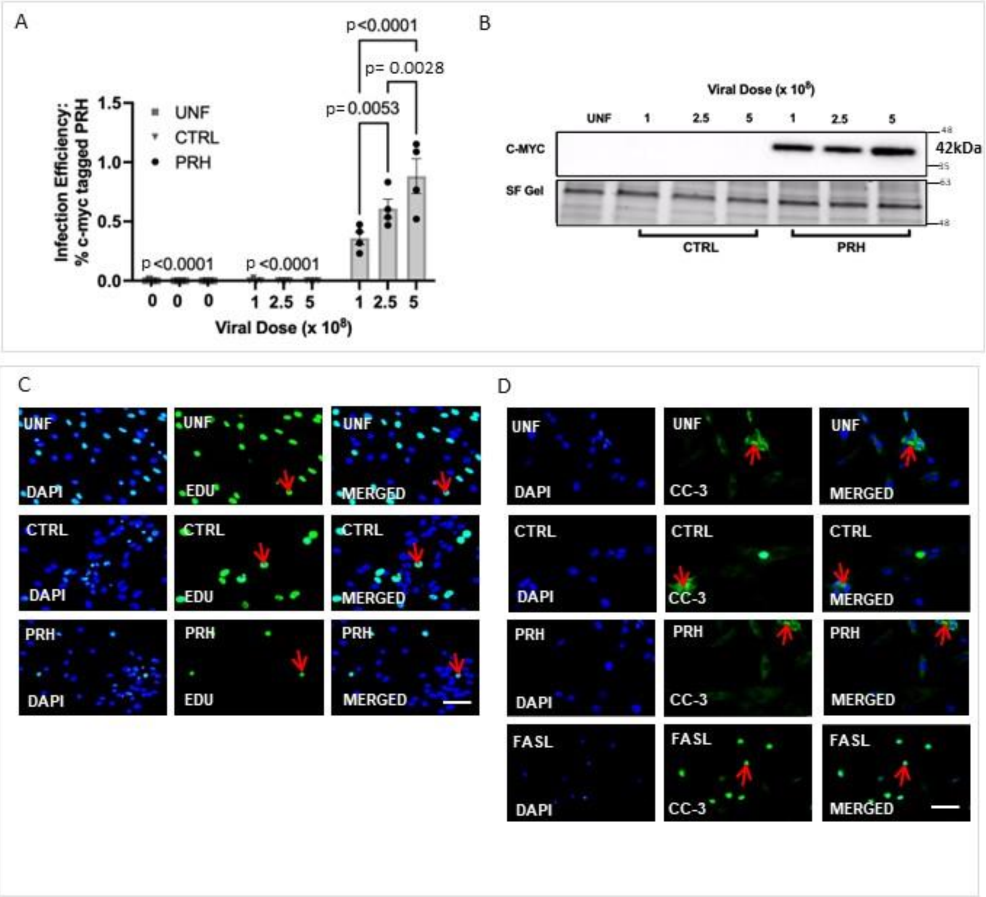

Online Figure S1: Overexpression of PRH S163C:S177C in HSV VSMCs.

HSV VSMCs were either infected with 1 x 10[8] pfu/mL, 2.5 x 10[8] pfu/mL or 5 x 10[8] pfu/mL Ad: Control or Ad: PRH S163C:S177C or were left uninfected**.** (**A**) Densitometric analysis of c-myc-tagged PRH S163C:S177C protein. Kruskal-Wallis ANOVA followed by Dunn's multiple comparison test, n=4. (**B**) Representative Western blot of c-myc-tagged PRH S163C:S177C protein. Stain-free bands served as a loading control. Approximate molecular weight of detected band indicated in kDa. (**C**) Representative images of Click-iT EdU imaging assay. Positive cells are green, and examples are indicated with red arrows; all nuclei are stained blue with DAPI. Scale bar indicates 50µm and applies to all panels. (**D**) Representative images of immunocytochemistry for cleaved caspase-3 (CC-3). Positive cells (white arrow) are green, and all nuclei are stained blue with DAPI. Scale bar measures 25 µm and applies to all panels. CC-3 denotes cleaved caspase-3.

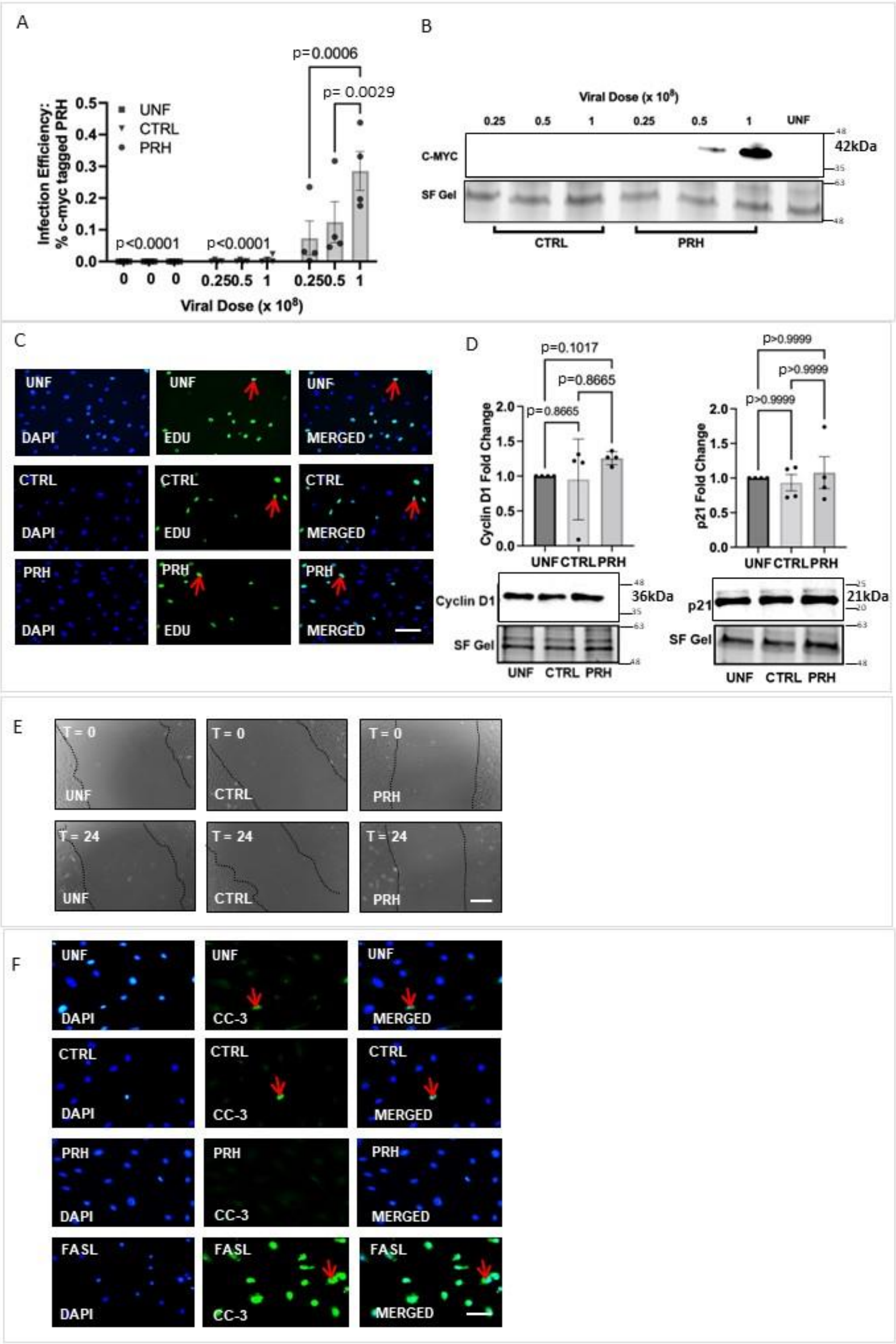

Online Figure S2: Overexpression of PRH S163C:S177C in HSVECs. HSVECs were either infected with 1 x 10^8 pfu/mL, 2.5 x 10^8 pfu/mL or 5 x 10^8 pfu/mL Ad: Control or Ad: PRH S163C:S177C or were left uninfected. (**A**) Densitometric analysis of c-myc-tagged PRH S163C:S177C protein. Kruskal-Wallis ANOVA followed by Dunn's multiple comparison test, n=4 (**B**) Representative Western blot of c-myc-tagged PRH S163C:S177C protein. Stain-free bands served as a loading control. Approximate molecular weight of detected band indicated in kDa. (**C**) Representative images of Click-iT EdU imaging assay. Positive cells are green, and examples are indicated with red arrows; all nuclei are stained blue with DAPI. Scale bar indicates 50µm and applies to all panels. (**D**) Densitometric quantification of cyclin D1 and p21 expression by Western blotting; data was normalised by stain-free bands and expressed as a fold change from uninfected control. (**E**) Representative images of scratch wound assay. Dashed line indicates wound edge. T0 = 0 hr; T24 = 24 hr. (**F**) Representative images of cleaved caspase-3 assay. Positive cells are green, and examples are indicated with red arrows; all nuclei are stained blue with DAPI. Scale bar indicates 50µm and applies to all panels.

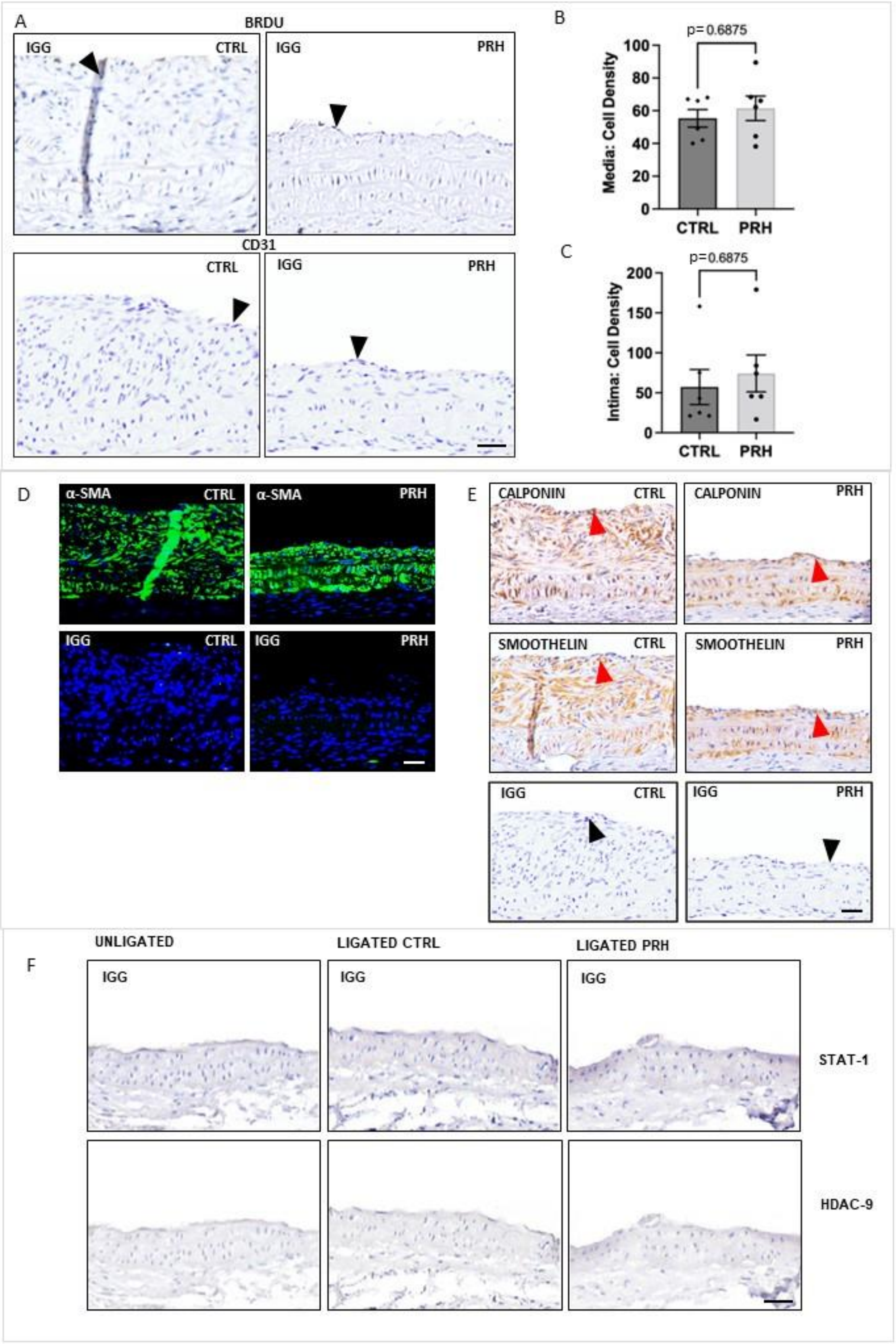

Online Figure S3: Overexpression of PRH S163C:S177C did not alter VSMC phenotype in vivo. Carotid arteries were ligated in C57B/L6 mice to induce intimal thickening then infected with Ad: Control or Ad PRH S163C:S177C. (A) Representative images of non-immune IgG for BrdU and CD31 protocols. Quantification of cell density in the medial (B) and intimal (C) layers 28 days after ligation. Data is expressed as the number of cells per mm2 of neointima. Representative images of immunofluorescence for αSMA and non-immune IgG (D), 28 days after ligation. αSMA -positive cells are stained green (some are indicated with red arrows) and the nuclei of all cells are stained blue/grey with DAPI. White dashed line indicates the intima-media boundary. (E) Representative images of immunofluorescence for calponin, smoothelin and non-immune IgG 28 days after ligation. Smoothelin- or calponin-positive cells are stained brown (some are indicated with red arrows); the nuclei of all cells are stained blue/grey with haematoxylin. Black dashed line indicates intimal: medial boundary. Scale bar represents 25 µm and applies to all panels. (F) Representative images of non-immune IgG for STAT-1 and HDAC-9 protocols. Students t-test and Mann-Whitney test, n=4. Error bars represent standard error of mean (SEM).

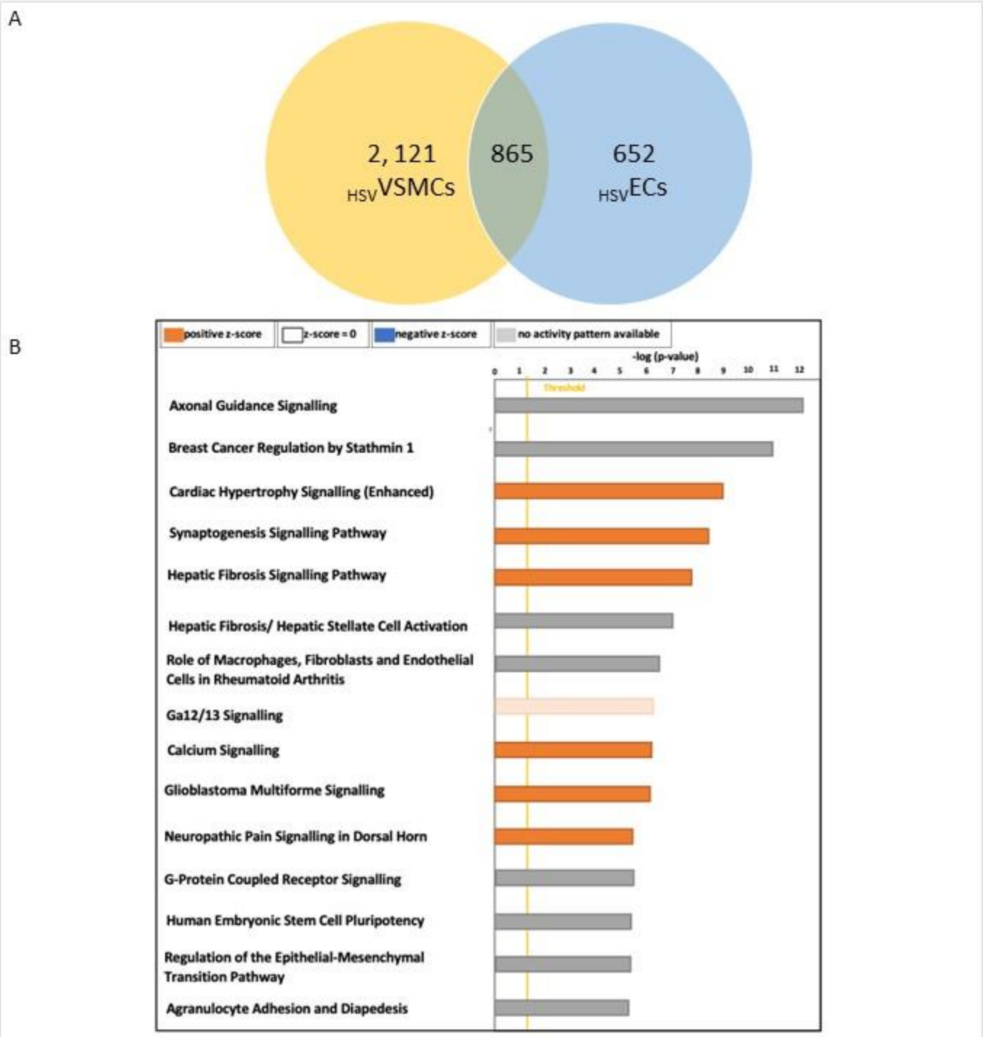

C

Gene	Protein	Function	Log_{10} Fold Change	-log 10 p-value
**HDAC-9**	Histone Deacetylase 9	transcription regulator/ repressor	-1.730	5.14E-05
**STAT-1**	Signal Transducer and Activator of Transcription 1	transcription factor	-1.244	6.54E-03
**CEBPD**	CCaAT Enhancer Binding Protein delta	transcription factor	-1.030	2.31E-02
**HAND-1**	Heart and Neural Crest Derivatives Expressed 1	transcription factor	6.923	1.48E-05
**NFATC-1**	Nuclear Factor of Activated T Cells 1	transcription factor	1.920	2.80E-05

Online Figure S4: Top-enriched canonical pathways of PRH S163C:S177C-regulated genes. (**A**) Venn diagram of PRH S163C:S177C-regulated DEGs. The previously filtered datasets were uploaded into the 'Compare Data tool' for further filtering of VSMC-specific and PRH S163C:S177C-specific DEGs. Dataset A: 2,121 DEGs that were exclusively PRH S163C:S177C-regulated in VSMCs. Dataset B: 652 DEGs were identified as exclusively PRH S163C:S177C-regulated in HSVECs. 865 DEGs were shared between the two cell types. (**B**) The significant Canonical Pathways for the dataset that were identified are displayed along the y-axis. By default, the x-axis displays the -log of the p-value which is calculated using the right-tailed Fisher's Exact Test, such that larger bars equate to increased significance. The yellow line indicates the threshold value. The orange bars in the bar chart indicate predicted pathway activation or predicted inhibition, respectively, via another statistic: the z-score. Grey bars indicate pathways for which no prediction can be made due to insufficient evidence in the Knowledge Base for confident activity predictions across datasets. (**C**) DEGs prioritised from the canonical pathways approach. P-value for inclusion here is ≤ .05. P- value is calculated using Fischer's exact test indicates the probability of the involvement of the genes in the dataset in either 'proliferation' and 'migration'. The direction of the fold change represents the upregulation (+) or downregulation (-) of a specific DEG. DEGs; Differentially expressed genes.

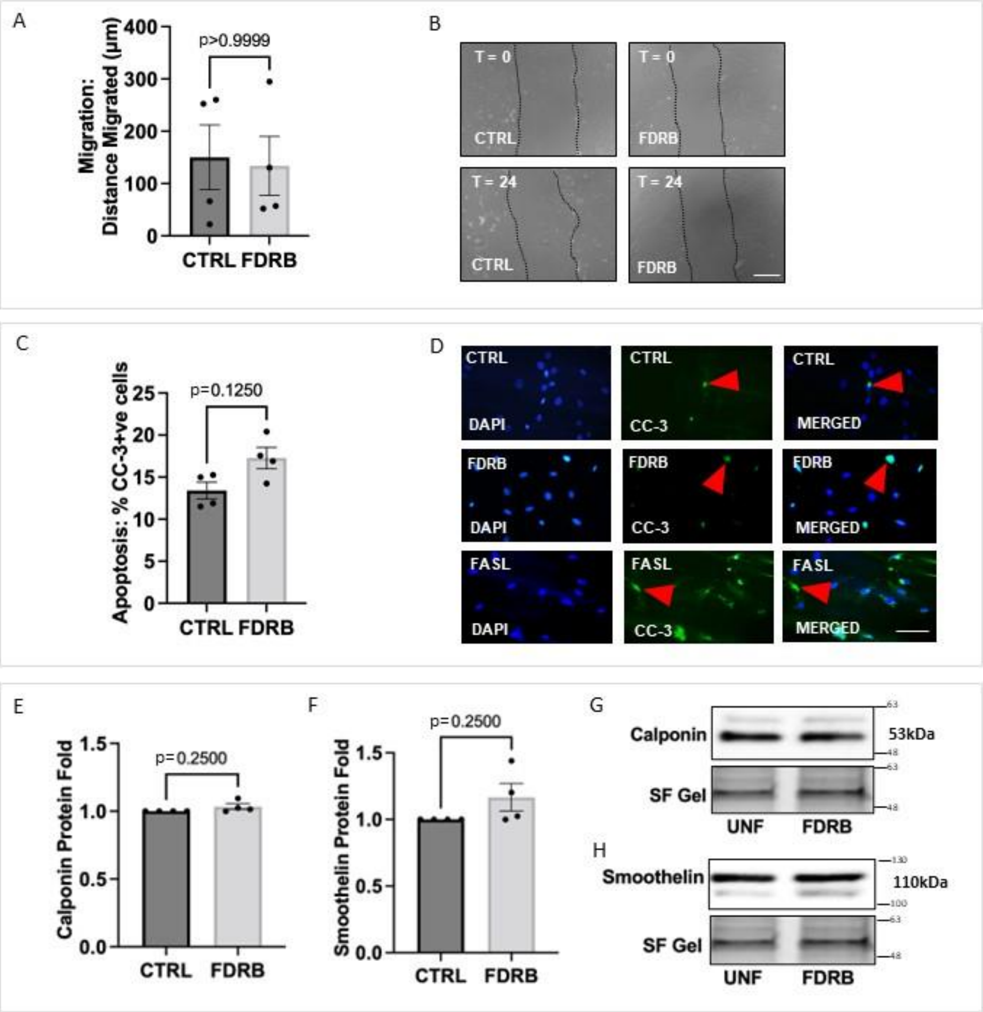

Online Figure S5: Fludarabine reduced VSMC proliferation without affecting proliferation, migration and VSMC phenotype. HSV-VSMCs were cultured with 50 µM fludarabine in DMSO or DMSO control alone. (**A**) Migration was quantified in µm; (**B**) Representative images of scratch wound assay. Dashed line indicates wound edge. T0 = 0 hr; T24 = 24 hr. (**C**) The rate of apoptosis was quantified and expressed as the percentage of cleaved caspase-3-positive cells. (**D**) Representative images of cleaved caspase-3 assay. Positive cells are green, and examples are indicated with red arrows; all nuclei are stained blue with DAPI. Quantification of calponin (**E**) and smoothelin (**F**) protein expression by Western blotting. Data was normalised by stain-free bands and expressed as a fold change from uninfected control. Representative Western blots for calponin (**G**) and smoothelin (**H**) proteins. Stain-free bands served as a loading control. Approximate molecular weights are indicated on the right in kDa. Scale bar indicates 50µm and applies to all panels. Students t-test and Mann-Whitney test, n=4. Error bars represent standard error of mean (SEM).

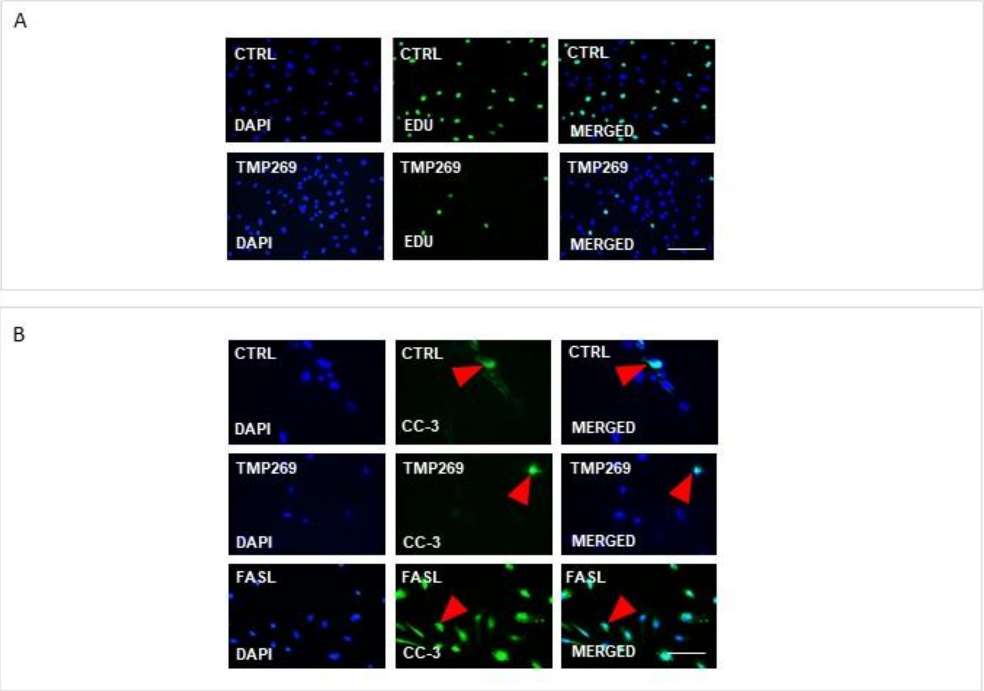

Online Figure S6: Fludarabine reduced VSMC proliferation without affecting proliferation, migration and VSMC phenotype. HSV VSMCs were cultured with 50 nM TMP269 in DMSO or DMSO control alone. (**A**) Representative images of Click-iT EdU imaging assay. Positive cells are green, and examples are indicated with red arrows; all nuclei are stained blue with DAPI. (**B**) Representative images of cleaved caspase-3 assay. Positive cells are green, and examples are indicated with red arrows; all nuclei are stained blue with DAPI. Scale bar indicates 50μm and applies to all panels.

Figure 1F – p21 protein

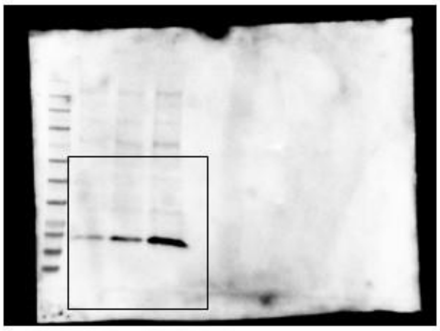

Figure 1G – cyclin D1 protein

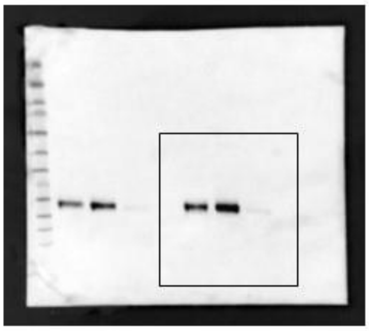

Figure 1M – calponin protein

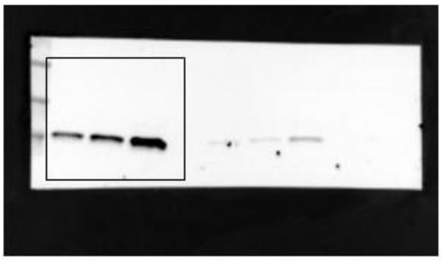

Figure 1N – smoothelin protein

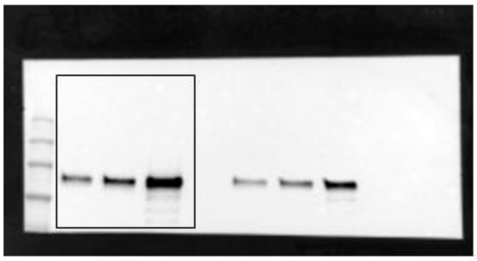

Figure 2D – c-myc-tag

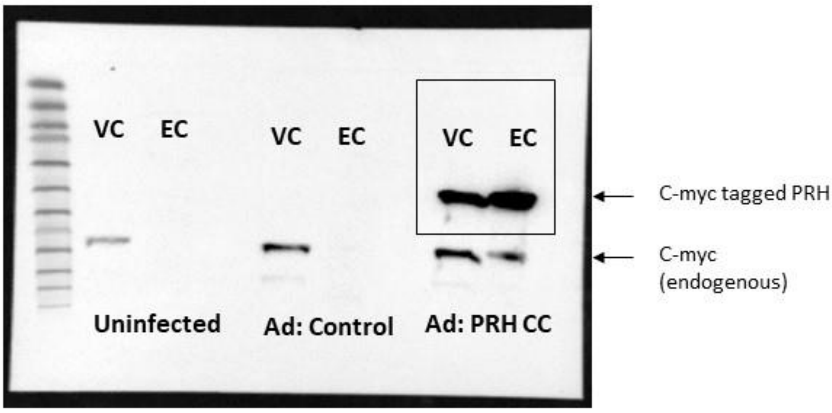

Online Figure S7: Uncropped representative Western blots from main figures.

Figure 4F – STAT1 protein

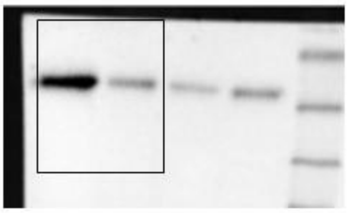

Figure 4B –STAT1 protein

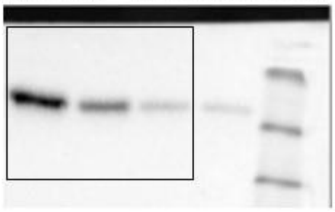

Figure 4I – p21 protein

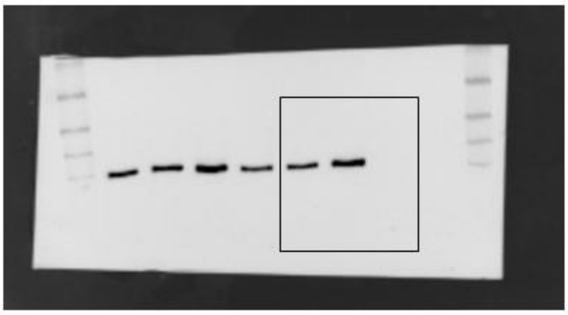

Figure 4J – cyclin D1 protein

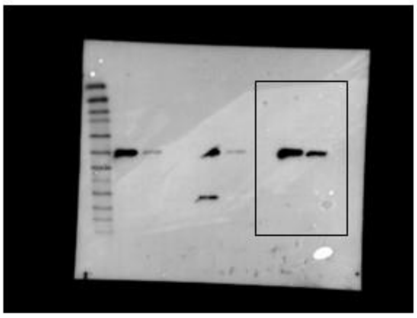

Figure 5C – STAT-1 protein

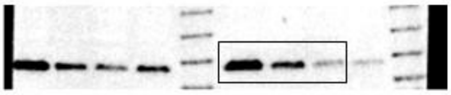

Figure 5K – cyclin D1 protein

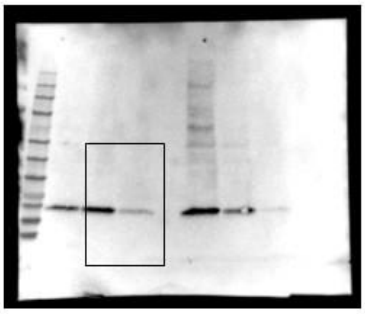

Figure 5C – HDAC-9 protein

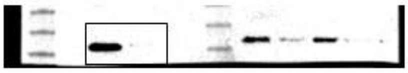

Figure 5R – smoothelin protein

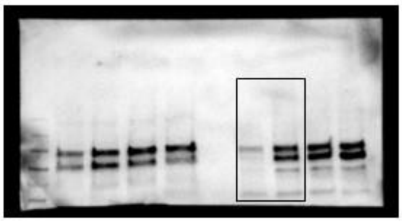

Figure 5Q – calponin protein

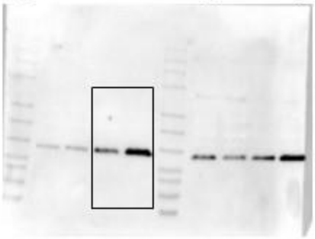

Online Figure S8: Uncropped representative Western blots from main figures.

Supp Figure IB – c-myc-tag

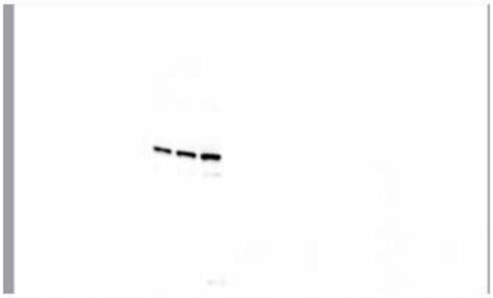

Supp Figure IIB – c-myc-tag

Supp Figure IID – p21 protein

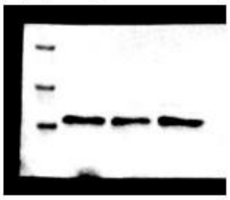

Supp Figure IID – cyclin D1 protein

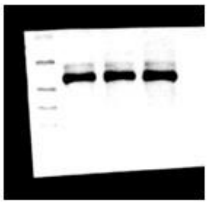

Supp Figure VG – calponin protein

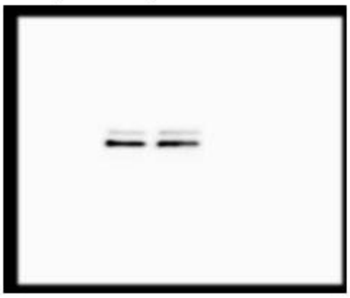

Supp Figure VH – smoothelin protein

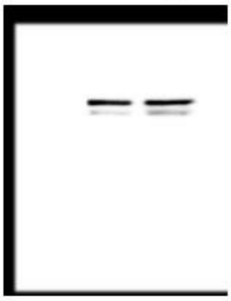

Online Figure S9: Uncropped representative Western blots from supplementary figures.
